# New Tricholidic Acid Triterpenoids from the Mushroom *Tricholoma ustaloides* Collected in an Italian Beech Wood

**DOI:** 10.3390/molecules28093864

**Published:** 2023-05-04

**Authors:** Gianluca Gilardoni, Francesca Negri, Paola Vita Finzi, Faiq H. S. Hussain, Giovanni Vidari

**Affiliations:** 1 Departamento de Química, Universidad Técnica Particular de Loja (UTPL), Loja 1101608, Ecuador; 2 Dipartimento di Chimica, Università degli Studi di Pavia, Via Taramelli 10, 27100 Pavia, Italy; 3 Department of Medical Analysis, Faculty of Applied Science, Tishk International University, Erbil 44001, Iraq

**Keywords:** *Tricholoma ustaloides*, Tricholomataceae (Basidiomycota), lanostane triterpenoids, tricholidic acid derivatives, saponaceolides, tricholomenyn, cytotoxicity

## Abstract

The secondary metabolites produced by *Tricholoma ustaloides* Romagn., a mushroom species belonging to the large *Tricholoma* genus (Basidiomycota, Tricholomataceae), are unknown. Therefore, encouraged by the interesting results obtained in our previous chemical analyses of a few *Tricholoma* species collected in Italian woods, we aimed to investigate the secondary metabolites of *Tricholoma ustaloides*. The chemical analysis involved the isolation and characterization of secondary metabolites through an extensive chromatographic study. The structures of isolated metabolites, including the absolute configuration, were established based on a detailed analysis of MS, NMR spectroscopic, optical rotation, and circular dicroism data, and on comparison with those of related compounds reported in the literature. Two novel lanostane triterpenoids, named tricholidic acids B and C, together with triglycerides, a mixture of free fatty acids, five unidentified metabolites, and the known rare saponaceolides F and J, tricholidic acid, and tricholomenyn C, were isolated from an EtOAc extract of fruiting bodies of *Tricholoma ustaloides* that were collected in an Italian beech wood. This is the second example of isolation of tricholidic acid derivatives from a natural source. Saponaceolides F and J exhibited high cytotoxicity (IC_50_ values ≤ 10 μM) against a panel of five human cancer cell lines. The toxicity against myeloid leukemia (HL-60), lung cancer (A-549), hepatocellular cancer (HepG2), renal cancer (Caki-1), and breast cancer (MCF-7) cells was higher than that shown by the very well-known cytotoxic drug cisplatin.

## 1. Introduction

The family Tricholomataceae, of the order Agaricales (Basidiomycota), comprises numerous genera. Among them, the genus *Tricholoma* (Fr.) Staude [1], including more than 250 species, is the largest one. All the known species are gilled mushrooms, mainly ectomycorrhizal with trees in the Pinaceae, Betulaceae, and Fagaceae families. They have a worldwide distribution [2] and are mainly found in temperate and subtropical zones in both the southern and northern hemispheres. The center of species richness appears to be in North America, from where more than 100 species have been reported [3], while 63 to 88 species are listed from Europe [4,5,6]. Several species are also described or reported from China, Japan, New Zealand, and Australia.

The taxonomy and phylogeny of the genus *Tricholoma* have been based, up to recent times, mainly on fungal morphological characters; however, the use of molecular methods based on nuc rDNA internal transcribed spacer ITS1-5.8S-ITS2 (ITS) sequences is becoming more and more important to analyze the diversity and distribution of several species [7,8,9]. Thus, phylogenetic analyses using nuclear and mitochondrial gene sequences have often proved that mushrooms collected with the same name in different places of the same country/continent [10,11] or in distant continents [8,10] must instead be placed in distinct clades/subclades or even different taxa. For example, *Tricholoma pardinum* (Pers.) Quél. was reported from the eastern Himalaya and adjacent areas, largely based on similar morphology with European samples. However, further detailed analyses of the morphology and DNA sequences indicated that two independent new species occur in southwestern China, namely, *T. highlandense* and *T. sinopardinum* [10]. The possible existence of different taxa may also explain the discrepancies in the chemical contents found in mushrooms known with the same name but growing in different habitats. The patterns of secondary metabolites isolated from *T. pardinum* collected in Germany and in Italy are a striking example of such differences. Lanostane triterpenoids [12], nor-terpenoids [13], bis-sesquiterpenoids [14], and polyketide-amino acid derivatives [15] were isolated from wild specimens of *T. pardinum* collected in southwestern Germany; in contrast, enyne polyols and ketols, together with 3-formylindoles, were isolated from wild specimens of *T. pardinum* collected in Italy [16].

Although a few species are considered toxic, such as *T. equestre*, several *Tricholomas* are highly valued due to their medicinal properties or as culinary delicacies. Moreover, *Tricholoma* fruiting bodies contain a variety of biological components, often with unusual structures, that include terpenoids, sterols, polyketide derivatives, aromatic acids, phenolic derivatives, and nitrogenous compounds [17]. In addition, a wide variety of biological activities have been determined for most *Tricholoma* metabolites, including cytotoxic and antimicrobial properties, neurite outgrowth stimulation effects, acetylcholinesterase inhibitory activity, etc. [17].

Our previous studies on *Tricholomas* have led to the isolation of different secondary metabolites with unprecedented structures, namely, cytotoxic C-30 saponaceolide terpenoids A–D, F, H, and T from *Tricholoma saponaceum* (Fr.) Kummer [18,19,20]; diterpenoid trichoaurantiolides A–D from *T. aurantium* (Schff.: Fr.) Ricken [21,22]; antimitotic enyne-geranyl-cyclohexenones, tricholomenyns A–E, from *T. acerbum* (Bull.: Fr.) Quél [23,24]; an acetogenin cyclopentene-dione, columbetdione, from *T. columbetta* (Fr.) Quél [25]; indole derivatives from *T. sciodes* and *T. virgatum* [26]; and four polioxygenated enynes from *T. pardinum* (Pers.) Quél [16]. In view of these interesting findings, and in continuation of our investigations on the contents of *Tricholoma* species growing in Italy [16], herein we report the results of the first phytochemical study of *Tricholoma ustaloides* Romagn., which was collected in an Italian beech wood. This mushroom, although generally rare, has a widespread distribution in Europe, where it typically grows in small groups from late summer to late autumn in association with oak, chestnut, hornbeam, and beech trees. According to Heilmann-Clausen and Christensen [7], *T. ustaloides* can be distinguished by the different habitats and molecular and morphological characters from similar mushrooms included in the section *Genuina* of *Tricholomas*.

**Figure 1:**
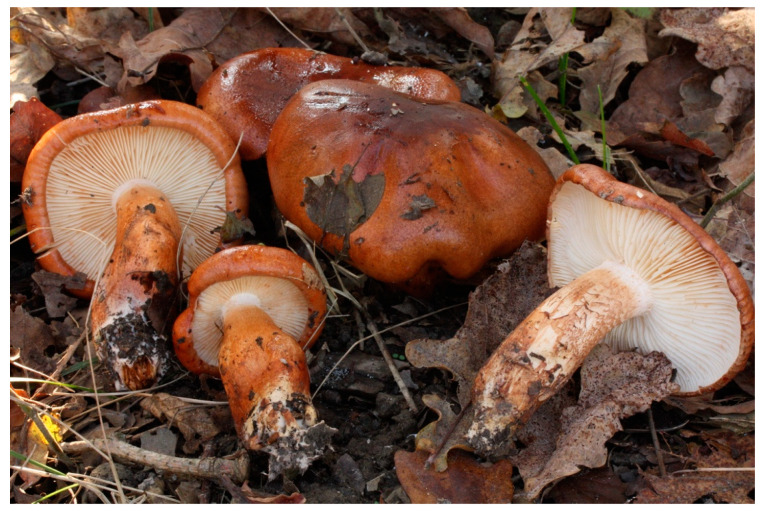
Specimens of *Tricholoma ustaloides* collected in a wood of Northern Italy (Photo by Teresio Restelli, Gruppo Micologico Pavese).

*T. ustaloides* (Figure 1) is recognized for a red-brown or chestnut-brown cap with a paler margin and a shiny viscous and glutinous cuticle when moist. The gills are crowded together, attached to the stipe, white or a light ochraceous yellow, with dark brown stains when old or bruised. The stipe is roughly spindle-shaped, with red-brown fibrils and a sharply defined pseudo-annular white zone at the stalk apex, especially in more mature specimens, which is a characteristic of the species. The flesh is white or cream, with a smell like that of watermelon peel or cucumber and an immediately perceived flour-bitter taste, especially in the cuticle. The mushroom is considered inedible in Europe [27], due to the unpleasant smell and taste; however, no toxic or cytotoxic effects have been reported so far and the mushroom is even consumed by some inhabitants of Mexico [28].

Our phytochemical investigation of an aqueous methanol sub-extract of an EtOAc extract of *T. ustaloides* fruiting bodies led to the isolation of two C-30 terpenoids, saponaceolides J (**1**) [29] and F (**2**) [20,30], three cyclic lactone-containing lanostane triterpenoids, tricholidic acid (**5**) [31] and tricholidic acids B (**6**) and C (**7**), together with tricholomenyn C (**8**) [24] (Figure 1), mixtures of triglycerides and free fatty acids, and five unidentified compounds. Triterpenoids **6** and **7** are novel compounds. The structures with absolute configurations were established by extensive MS, 1D, and 2D NMR spectroscopic methods, as well as optical rotation and CD data and comparison with the literature. Moreover, the cytotoxic activities of compounds **1**, **2**, **5**–**7** were evaluated against myeloid leukemia (HL-60), lung cancer (A-549), hepatocellular cancer (HepG2), renal cancer (Caki-1), and breast cancer (MCF-7) cells. This paper reports the isolation, structural elucidation, and cytotoxicity of the isolates.

## 2. Results

### 2.1. Mushroom Extraction and Isolation of Secondary Metabolites

**Scheme 1:**
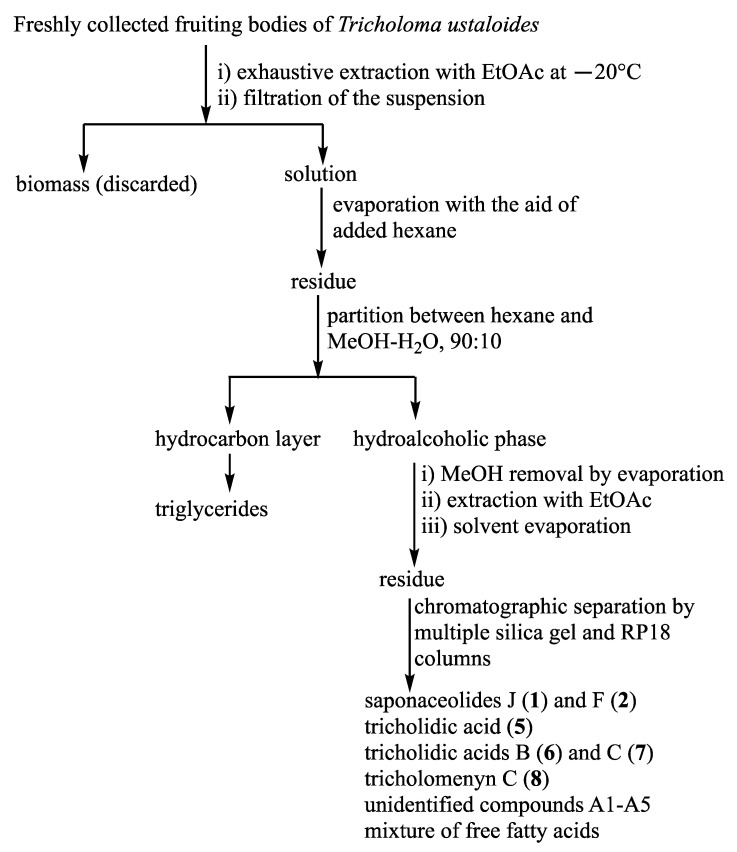
Extraction and isolation of secondary metabolites from *Tricholoma ustaloides*.

Extraction of fruiting bodies has always been a critical step in the isolation of fungal metabolites because an incorrect procedure may readily lead to the formation of artifacts. In fact, enzymatic reactions and/or the alcoholic solvent often used for the extraction may cause extensive compound degradation [32]. To avoid these problems, according to an optimized procedure, freshly collected fruiting bodies of *T. ustaloides* were frozen at −20 °C, chopped still frozen, and extracted with EtOAc in the cold. After biomass separation by filtration, the solution was taken to dryness by solvent removal under a vacuum at ≤35 °C. Evaporation of the last part of the extract was facilitated by multiple additions of hexane. This expedient also prevented a dangerous concentration of AcOH in the extract. Acetic acid was inevitably produced by the contact of EtOAc with the large amount of H_2_O (about 90% *w*/*w*) contained in the fruiting bodies. The residue was then partitioned between hexane and MeOH-H_2_O, 9:1. This procedure led to the almost quantitative extraction of triglycerides (NMR) and other non-polar compounds by the hydrocarbon phase. The residue from the aqueous methanolic layer was finally fractioned in the standard way by multiple open air and medium pressure liquid chromatographic (MPLC) separations on silica gel and reversed-phase columns, to give pure (NMR and TLC) compounds **1**, **2**, **5**–**7**, five unidentified metabolites A1–A5, and a mixture of common free saturated and unsaturated free fatty acids (NMR) which were not analyzed. The extraction and isolation process are briefly illustrated in Scheme 1.

### 2.2. Structure Determination

**Figure 2:**
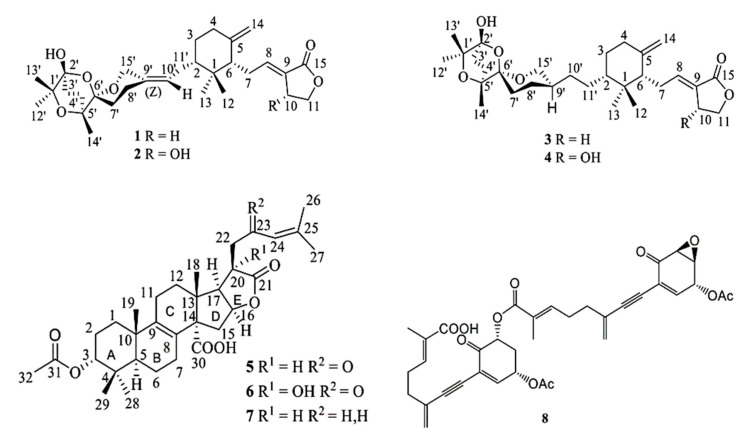
Structures of saponaceolides J (**1**), F (**2**), B (**3**), A (**4**), tricholidic acids (**5**), B (**6**), C (**7**), and tricholomenyn C (**8**).

Compound **1** was isolated as a white powder. The counting of protons and carbons occurring in the NMR spectra, together with the ion [M+NH_4_]^+^ at *m*/*z* 518 (C_30_H_48_NO_6_^+^) in the DCI-MS (NH_3_) spectrum, indicated that the molecular formula was C_30_H_44_O_6_. The ^1^H and ^13^C NMR data of the tricyclic spiroacetal unit and the sesquiterpenoid moiety from C1 to C15 of compound **1** were almost identical to those of saponaceolide B (**3**) [19,30] with the exception of the signals characteristic of an additional trisubstituted double bond [*δ*_C_ 134.4 (s, C9′) and 123.0 (d, C10′), and *δ*_H_ 5.16 (1 H, t, *J* = 7.4 Hz, H9′)]. The HMBC spectrum suggested that the double bond was located between C9′ and C10′ based on the correlations of H15′a (*δ*_H_ 4.45, 1 H, d, *J* = 13.5) and H15′b (*δ*_H_ 4.28, 1 H, d, *J* = 13.5) with C9′, and those of H8′a (*δ*_H_ 2.11, 1 H, m) and H8′b (*δ*_H_ 2.02, 1 H, m) with C9′. Moreover, the presence of a double bond between C9′ and C10′ resulted in the upfield shift of C15′ (*δ*_C_ 60.0, t) and a downfield shift of C8′ (*δ*_C_ 27.7, t), compared to the corresponding signals in the ^13^C NMR spectrum of the dihydroderivative, saponaceolide B (**3**) [19,33]. Thus, the structure of compound **1** (Figure 2) is that of saponaceolide J, previously isolated from *T. terreum* [29]. The NMR data and the optical rotation of **1** corresponded with those in the literature [29].

Compound **2** was isolated as a white powder. The counting of protons and carbons occurring in the NMR spectra, together with the ion [M + NH_4_]^+^ at *m*/*z* 534 (C_30_H_48_NO_7_^+^) in the DCI-MS (NH_3_) spectrum, indicated that the molecular formula was C_30_H_44_O_7_, differing from compound **1** by one oxygen atom. The ^1^H-NMR and ^13^C spectra of **2** exhibited the same features as saponaceolide A (**4**) [18], including the presence of a hydroxy-substituted γ-lactone, which was indicated by the signals at *δ*_H_ 5.05 ppm (1 H, br d, *J* = 5.5 Hz, H10) and *δ*_C_ 66.3 (d, C10). The main differences between the NMR spectra of compounds **2** and **4** were the appearance in the spectra of **2** of an additional olefinic proton at *δ*_H_ 5.16 (1 H, t, *J* = 7.0 Hz, H9′) and an additional double bond [*δ*_C_ 134.7 (s, C9′) and 123.0 (d, C10′)]. These data indicated the presence of a double bond between the carbons C9′ and C10′ of saponaceolide **2**, as in saponaceolide J (**1**). Hence, the structure of compound **2** was determined to be saponaceolide F (Figure 2). The NMR data and the optical rotation of **2** nicely corresponded to those of the metabolite previously isolated from *T. saponaceum* [20,30], although different solvents were used for the measurements [30].

Compound **5** was isolated as a crystalline compound, mp 208–212 °C, [α]_D_^23^ = −128.1 (*c* = 3.6 mg/mL, CH_2_Cl_2_). The formula C_32_H_44_O_7_ was deduced from the ion [M+NH_4_]^+^ at *m*/*z* 558 (C_32_H_48_NO_7_^+^) in the DCI-MS (NH_3_) spectrum, together with the counting of protons and carbons occurring in the ^1^H and ^13^C NMR spectra (Table 1), respectively. The ^1^H NMR spectrum (Table 1) of **5** displayed four singlets (3H each) at *δ*_H_ 0.87 (H_3_28), 0.92 (H_3_18), 0.94 (H_3_19), and 1.04 (H_3_29), attributable to four tertiary methyls; two doublets (3 H each, *J* = 1.2 Hz) at *δ*_H_ 1.93 (H_3_26) and *δ*_H_ 2.16 (H_3_27), assignable to two vinylic methyls; a sharp singlet (3 H) at *δ*_H_ 2.05, assignable to an acetate methyl (H_3_32); a triplet at *δ*_H_ 4.67 (1 H, *J* = 2.5 Hz), assignable, through a ^3^*J* correlation, to proton H3 geminal to an acetoxy group; and a septuplet (1 H, *J* = 1.2 Hz) at *δ*_H_ 6.12, attributable to an olefinic proton (H24), that was coupled to the vinylic methyls H_3_26 and H_3_27 and flanked by an α,β-unsaturated ketone carbonyl group (*δ*_C_ 196.9, s, C23). The COSY spectrum of **5** revealed the presence of an extended coupled proton spin system, H_2_22 [*δ*_H_ 2.82 (1 H, dd, *J* = 18.6, and 9.1 Hz, H_22a_) and *δ*_H_ 3.15 (1 H, dd, *J* = 18.6 and 4.0 Hz, H_22b_)]-H20 [*δ*_H_ 3.46 (1 H, td, *J* = 8.9 and 4.0 Hz)]-H17 [*δ*_H_ 2.87 (1 H, dd, *J* = 8.7 and 6.7 Hz)]-H16 [*δ*_H_ 5.15 (1 H, ddd, *J* = 7.5, 7.0, and 4.1 Hz)]-H_2_15 [*δ*_H_1.97 (1 H, dd, *J* = 13.9 and 4.0 Hz, H_15a_) and *δ*_H_ 2.73 (1 H, dd, *J* = 13.9 and 7.5 Hz, H_15b_)]. Three additional carbons resonated as singlets at *δ*_C_ 178.6, 177.6, and 170.7 in the ^13^C NMR spectrum of **5**, (Table 1), which were assigned, respectively, to the carbonyls of a γ-lactone (C21), a carboxylic acid (C30), and an acetate group (C31). The IR bands at 1750, 1730–1700, 1690, and 1626 cm^−1^ sustained the presence of these functional groups, and the carboxylic function was fully confirmed by the formation of a methyl ester (*δ*_H_ 3.70, 3 H, s, OMe) upon exposure of **5** to CH_2_N_2_. Other characteristic signals in the ^13^C NMR spectrum of compound **5** appeared at *δ*_C_ 83.7 (d, C16) and 77.4 (d, C3), 127.3 (0, C8) and 142.0 (0, C9), 122.9 (d, C24) and *δ*_C_ 157.2 (s, C25). They were assigned, respectively, to a lactone and an acetoxy methine carbon, a tetrasubstituted olefin carbon, and a trisubstituted olefin carbon conjugated to the ketone CO (*δ*_C_ 196.9, s, C23). Moreover, H_2_15 showed a ^3^*J* correlation with the carboxylic carbon (*δ*_C_ 177.6, s, C30), whereas H16 (*δ*_H_ 5.15) and H20 (*δ*_H_ 3.46) exhibited a ^3^*J* and a ^2^*J* correlation, respectively, with the γ-lactone carbonyl carbon (*δ*_C_ 178.6, s, C21).

These data clearly suggested that compound **5** was a pentacyclic lactonic acid lanostane triterpenoid, bearing an α-oriented acetoxy group at C3. In fact, the almost identical physical and spectroscopic data of compound **5** with the literature led us to identify compound **5** with tricholidic acid (Figure 2), whose structure was firmly established by an X-ray crystallographic analysis [31]. Moreover, the negative sign of the CD curve of **5** (Δe = −2.08 at about 225 nm), which was attributed to the lactone ring [31], indicated the same absolute configuration as tricholidic acid [31] (Figure 2).

Compound **6** was isolated as colorless powder, [α]_D_^22^—102.3 (*c* 7.7 mg/mL, CH_2_Cl_2_). The MW = 556 and the molecular formula C_32_H_44_O_8_ were established by the ion [M + NH_4_]^+^ at *m*/*z* 574 (C_32_H_48_NO_8_^+^) in the DCI-MS (NH_3_) spectrum and the NMR data (Table 1). The ^1^H and ^13^C NMR spectra of compounds **5** and **6** were very similar (Table 1), as well as those of the corresponding methyl esters, except for the signals attributable to the moiety C17-C20-C22. In fact, in the ^1^H NMR spectrum of compound **6**, both the methylene protons H_2_22 [*δ*_H_ 2.86 (1 H, d, *J* = 18.0 Hz, H_22a_) and *δ*_H_ 3.21 (1 H, d, *J* = 18.0 Hz, H_22b_)] and the methine H17 [*δ*_H_ 2.72 (1 H, d, *J* = 6.3 Hz)] showed no coupling with H20. This finding suggested the presence of a substituent at C20. The strong band of chelated hydroxy groups in the IR spectrum of compound **6** and the occurrence of a quaternary carbon at *δ*_c_ 76.6 (s) in the ^13^C NMR spectrum of **6** clearly indicated the presence of an OH group at C20. Accordingly, due to the β-effect, both the C17 and C22 signals in the ^13^C NMR spectrum of compound **6** were significantly shifted downfield in comparison with the corresponding signals in the spectrum of **5**. Thus, the structure of 20-hydroxytricholidic acid was attributed to **6**, which was named tricholidic acid B. The almost identical NMR data of compounds **5** and **6** from C1 to C14 (Table 1) indicated that they had the same relative configuration 3R*, 5R*, 10S*, 13R*, 14S*. About the relative configuration at C16, C17, and C20 stereocenters, the *cis* relationship between H16 and H17 was suggested by the vicinal coupling constant *J*_16,17_ of 6.3 Hz. On the other hand, H16 was shifted downfield by about 0.3 ppm in the ^13^H NMR spectrum of **6**, compared to the corresponding signal of **5**, whereas selective irradiation of H_3_18 produced a nOe effect on the signals of H_2_22. These data clearly indicated that the 20-OH group was *cis* to H16 and H17 and *anti* to H_3_18, suggesting the relative configuration 16R*, 17S*, 20R* (Figure 2). Finally, the negative sign of the ECD curve, like that of **5**, suggested the absolute configuration of **6** to be 3*R*, 5*R*, 10*S*, 13*R*, 14*S*, 16*S*, 17*R*, 20*R.*

Compound **7** was isolated as a sticky, pale yellow oil. It showed negative signs of the rotation and the Cotton effect; however, the values of [α]_D_^22^ and Δε could not be determined because of the paucity of material. The MW = 526 and the molecular formula C_32_H_46_O_6_ were established by the pseudomolecular ion [M+NH_4_]^+^ at *m*/*z* 544 (C_32_H_50_NO_6_^+^) in the DCI-MS (NH_3_) spectrum and the counting of protons and carbons occurring in the NMR spectra (Table 1). The IR spectrum revealed the presence of γ-lactone (1770 cm^−1^), ester (1730 cm^−1^), and carboxylic (1700 cm^−1^) groups. These functions gave rise to three singlets at *δ*_c_ 178.7, 170.6, and 177.2, respectively, in the ^13^C NMR spectrum of **7**. Moreover, the acid **7** afforded a methyl ester (*δ*_H_ 3.68, 3H, s, OMe) on treatment with CH_2_N_2_. With the aid of ^1^H, DEPT and HSQC spectra, the 46 proton and the 32 carbon resonances as displayed by the ^1^H NMR and ^13^C NMR spectra of **7** (Table 1) were ascribed to seven CH_3_, including two olefinic methyls [*δ*_c_ 17.8 (s, C26) and 25.7 (s, C27); *δ*_H_ 1.61 (3 H, br s, H_3_26) and 1.70 (3 H, br s, H_3_27)], nine methylenes, six methines, including one olefinic [*δ*_c_ 123.2 (d, C24); *δ*_H_ 5.10 (1 H, m, H24)] and two oxygenated ones [*δ*_c_ 77.6 (d, C3) and 83.2 (d, C16); *δ*_H_ 4.67 (1 H, t, *J* = 2.5 Hz, H3) and 5.06 (1H, m, H16)], and ten quaternary carbons, that included three carbonyl carbons [170.6 (s, C31), 177.2 (s, C30), and 178.7 (s, C21)] and three olefinic carbons [*δ*_c_ 128.1 (s, C8), 133.2 (s, C25), and 142.3 (s, C9)]. Analysis of these and other NMR features implied that triterpenoid **7** had the same structure as tricholidic acid (**5**), except for the absence of the unsaturated ketone CO at C23. In fact, in the ^13^C NMR spectrum of **7**, no carbonyl signal was observed in the range of *δ*_c_ 195–200, while a methylene carbon resonated at *δ*_c_ 22.0 (t, C23), bearing two geminal protons (H_2_23, *δ*_H_ 2.2–2.35, m) coupled (COSY) with the olefinic proton H24 [*δ*_H_ 5.10 (1 H, m)]. Moreover, the olefinic carbon C25 (*δ*_c_ 133.2, s) was shifted upfield compared to the corresponding signal (*δ*_c_ 157.2, s) occurring in the ^13^C NMR spectrum of **5**, due to the loss of conjugation with the CO. The change in electronic environment also caused the shielding (0.76 ppm) of H20 [δ_H_ 2.70 (1 H, m)] compared to the corresponding signal (*δ*_H_ 3.46) in the ^1^H NMR spectrum of compound **5**. Thus, the structure of 23-deoxotriholodic acid was assigned to triterpenoid **7** (Figure 2), which was named tricholidic acid C. Biosynthetic considerations, the cooccurrence in the same fruiting bodies, and the negative sign of the Cotton effect at about 220 nm suggested the absolute configuration of **7** to be the same as that of tricholidic acids **5** and **6** (Figure 2).

Compound **8** (Figure 2) was identified as tricholomenyn C by the identity of the physicochemical and NMR data within the literature [24] and comparison with an authentic sample.

### 2.3. Cytotoxicities (MTS Assays)

The cytotoxicity of isolated compounds **1**, **2**, **5**–**7** was evaluated using the MTS method [34]. For comparison, saponaceolides A (**4**) and B (**3**) were added to the text, whereas cisplatin was used as the reference cytotoxic compound. In a preliminary test, triterpenoids **5**–**7** showed weak cytotoxicity (IC_50_ > 25 μM) against human myeloid leukemia HL-60 cells, while IC_50_ values of saponaceolides **1**–**4** were in the range of 0.3–1.5 μM. Therefore, compounds **1**–**4** were also tested against lung cancer A-549, hepatoma Hep G2, renal cancer Caki-1, and breast cancer MCF-7 cells, while triterpenoids **5**–**7** were not tested against this cell panel. The determined cytotoxicity data (IC_50_ values) are reported in Table 2.

## 3. Discussion

The chemical constituents of the fruiting bodies of *T. ustaloides* were investigated for the first time. Six rare representative metabolites of different biogenesis were isolated from this mushroom, namely, dimeric sesquiterpenoid saponaceolides **1** and **2**, triterpenoids **5**–**7** with a modified lanostane structure, and tricholomenyn C (**8**) of mixed biogenesis. Saponaceolides J (**1**) and F (**2**) were previously isolated only once from nature, namely, from *T. terreum* [29] and from *T. saponaceum* [20,30], respectively. Thus, with the occurrence of **1** and **2** in *T. ustaloides*, examples of terpenoids with the saponaceolide skeleton have been found as characteristic metabolites of *Tricholoma* species placed in the sections *Contextocutis* (= sect. *Rigida*), *Terrea*, *Atrosquamosa*, and *Genuina* [4,7,17]. Tricholidic acid (**5**) was previously isolated only once, namely, from a *Tricholoma* species collected in Japan [31], which is probably identifiable as *T. albobrunneum* [11]; instead, tricholidic acids B (**6**) and C (**7**) are novel triterpenoids, isolated for the first time from *T. ustaloides* in this study. Interestingly, from a chemotaxonomic point of view, both *T. albobrunneum* and *T. ustaloides* belong to the section *Genuina* of *Tricholomas* [4,7]. This section also includes the species *T. imbricatum*, which produces the degraded lanostane triterpenoids tricholimbrins A and B, which are structurally related to tricholidic acids **5**–**7** [35]. Finally, this is the second finding in nature of tricholomenyn C (**8**), which was isolated for the first time by us from *T. acerbum* [24], which belongs to the section *Megatricholoma* [4,7].

The compounds **1**, **2**, **5**–**8** belong to families which, although distributed in different sections of *Tricholomas*, appear to be characteristic metabolites of the genus. In fact, there are no other examples of these types which have been isolated from mushrooms or other living organisms.

The cytotoxicity against five human cancer cell lines, determined by an MTS assay [34], revealed that saponaceolides J (**1**) and F (**2**) possessed high cytotoxicity (Table 2), confirming a biological property which appears to be common to the members of this family of secondary metabolites [18,19,20]. Preliminary results indicated that the cytotoxicity might be attributed to the presence of an α-alkylidene-γ-butyrolactone unit capable of undergoing a Michael reaction with biological nucleophiles such as L-cysteine or thiol-containing enzymes [36]. However, synthetic studies showed that a simplified structure, mimicking the right hand of the saponaceolide molecules and devoid of the dissymmetric tricyclospiroketal moiety, did not match the pattern of antitumor activities of the entire molecules. These results pointed out the need of the entire skeleton of saponaceolides for maintaining the specificity and potency of the cytotoxic activity [37]. Moreover, the comparison of the IC_50_ values of **1**–**4** (Table 2) suggested that an OH group on the γ-lactone ring of saponaceolides (**2** vs. **1** and **4** vs. **3**), as well as the introduction of a double bond between C9′ and C10′ (**1**/**2** vs. **3**/**4**), increased the cytotoxicity.

## 4. Materials and Methods

### 4.1. General Experimental Techniques and Procedures

Preparative chromatographic separations were carried out on open columns at atmospheric pressure (CC). The columns were manually packed with silica gel (Merck Kieselgel 60, 40–63 μm, Rahway, NJ, USA) or C_18_ reversed phase (Merck LiChroprep RP-18, 25–40 μm) powder, purchased from Sigma-Aldrich (St. Louis, MO, USA). Thin-layer chromatographic (TLC) analyses were conducted over glass-supported silica gel 60 (0.25 mm; GF_254_, Merck) or RP-18 (F254s, Merck) plates (Sigma-Aldrich). Spots on TLC plates were initially visualized under UV light (254 and 366 nm); subsequently, they were sprayed with a 0.5% solution of vanillin in H_2_SO_4_/ethanol 4:1, and finally heated by a hot gun until maximum color development. Semipreparative medium pressure liquid chromatographic (MPLC) separations were performed by an Isolera instrument (Biotage, Uppsala, Sweden), equipped with silica gel and RP-18 reversed phase cartridges and a dual wavelength UV detector. Reagent-grade solvents, purchased from Carlo Erba (Milan, Italy) or from Aldrich, were used for extraction and chromatographic separations. Optical rotation: PerkinElmer 241 polarimeter (Walthman, MA, USA); CD spectra: Jasco J-1500 CD spectrometer (Tokyo, Japan). IR spectra: PerkinElmer Paragon 100 PC FT-IR spectrometer (Walthman, MA, USA), on KBr disks. NMR spectra: Bruker AV300 spectrometer, at 300 (^1^H) and 75.47 MHz (^13^C), operating at 22 °C (Billerica, MA, USA). ^1^H NMR and ^13^C NMR chemical shifts are relative to signals of residual C*H*Cl_3_ (δ_H_ 7.25, singlet) and ^13^*C*DCl_3_ *δ*_C_ (77.0, central line of a triplet) in CDCl_3_ (Sigma-Aldrich, Steinheim, Germany); coupling constants (*J*) in Hz; multiplicity (=number of attached hydrogens) of each C-atom was determined by DEPT experiments; COSY, DEPT, and HSQC spectra, and NOE effects were recorded using standard pulse sequences. DCI-MS (NH_3_): Finnigan-MAT 822 mass-spectrometer.

### 4.2. Fungal Material

Fruiting bodies of *Tricholoma ustaloides* Romagn. (990 g) were collected at the end of September—beginning of October 2021 in a beech wood near Passo del Brallo (GPS coordinates: 44°73′ N, 9°28′ E; alt. about 950 m above sea level), in the Province of Pavia (Italy). The fungus was identified by Alfredo Gatti, vice-president of the Voghera Mycological Group. A sample specimen (accession code: TU001) was deposited at the Department of Chemistry, University of Pavia, Italy.

### 4.3. Extraction and Isolation

Fresh fruiting bodies were brought in jute bags to the laboratory about 2 h after the collection and quickly frozen at −20 °C. Subsequently, they were roughly chopped and soaked three times with EtOAc (1.5 L each time) at −20 °C for 2 h each time. The extracts were put together and evaporated under reduced pressure in a water bath at <35 °C until 1/10 the original volume. Hexane (400 mL) was added, and the solution was evaporated to half volume; an additional volume of hexane was added, and the procedure was repeated three more times. Eventually, the solution was taken to dryness to give a brown oily residue (4.18 g). The residue was partitioned between MeOH-H_2_O, 90:10 (200 mL), and hexane (30 mL) four times to give a crude hexane extract (1.2 g) containing triglycerides (NMR) and other non-polar compounds. After methanol removal by evaporation, the aqueous phase was diluted with brine (60 mL) and extracted exhaustively with EtOAc. After drying (Na_2_SO_4_ + MgSO_4_), evaporation of the organic phase under reduced pressure afforded a dense oily residue (2.8 g). This mixture was subjected to silica gel CC (300 g). Elution with a CH_2_Cl_2_-Me_2_CO gradient (10:1–1:1), followed by MeOH, afforded seven main fractions (I–VII). Preliminary TLC analysis of these fractions on silica gel and reversed phase plates revealed that I, VI, and VII contained complex mixtures of compounds including those occurring in less complex fractions II–V. Therefore, I, VI, and VII were not analyzed. Fraction II (130 mg) was separated by a semipreparative MPLC silica gel column (16 g). Elution with a CH_2_Cl_2_-Me_2_CO gradient (40:1–5:1) afforded unidentified compound A1 (51 mg) and saponaceolide J (**1**, 6.7 mg). Fraction III (180.3 mg) was separated by silica gel CC (19 g). Elution with CH_2_Cl_2_-Me_2_CO (10:1) gave subfractions III-1-III-4. Subfraction III-2 contained tricholidic acid C (**7**, 3.4 mg). Subfraction III-3 (104 mg) was fractioned by an RP-18 MPLC column with MeOH-H_2_O, 5:1, to yield unidentified compound A2 (27 mg) and saponaceolide F (**2**, 35 mg). Separation of subfraction III-4 (38.6 mg) by a MPLC RP-18 column (5 g) with MeOH-H_2_O, 5:1, yielded more A2 (5 mg) and subfraction III-4–1 (23 mg), which was further separated by chromatography on a silica gel column (5 g). Elution with CH_2_Cl_2_-Me_2_CO (8:1) afforded unidentified compounds A3 (5.5 mg) and A4 (15.2 mg). Fraction IV (100 mg) was separated by silica gel CC (11 g) with a gradient of CH_2_Cl_2_-Me_2_CO (8:1–6:1) to yield subfraction IV-1 (10.2 mg) and a mixture of free fatty acids (NMR) (42 mg) which was not analyzed. Purification of IV-1 by a semipreparative MPLC RP18 column (10g) with MeOH-H_2_O, 5:1, yielded tricholidic acid (**5**, 6 mg). Separation of fraction V (130 mg) by RP18 CC (15g) with MeOH-H_2_O, 5:1, afforded, in the following order, unidentified compound A5 (13.6 mg), tricholomenyn C (**8**, 10 mg), and tricholidic acid B (**6**, 32 mg).

#### 4.3.1. Saponaceolide J (**1**)

Colorless powder; [α]_D_^22^ + 36.4 (*c* 2.5 mg/mL, CH_2_Cl_2_); R_f_ 0.44 (silica gel TLC; CH_2_Cl_2_-Me_2_CO, 30:1); ^1^H NMR (300 MHz, CDCl_3_): *δ*_H_ = 6.70 (1H, m, H8), 5.16 (1 H, t, *J* = 7.4 Hz, H10′), 4.84 (1 H, q, *J* = 1 Hz, H14a), 4.45 (1 H, br d, *J* = 13.5 Hz, H15′a), 4.41 (1 H, q, *J* = 1 Hz, H14b), 4.39 (2 H, t, *J* = 7.5 Hz, H_2_11), 4.28 (1 H, br d, *J* = 13.5 Hz, H15′b), 2.87 (2 H, m, H_2_10), 2.65–0.8 (19 H, overlapped m’s, H_2_3, H_2_4, H_2_7, H_2_3′, H_2_4′, H_2_7′, H_2_8′, H_2_11′, H2, H6, HO2′), 1.30 (3 H, s, H_3_12′), 1.23 (3 H, s, H_3_13′), 1.08 (3 H, s, H_3_14′), 1.06 (3 H, s, H_3_12), 0.60 (3 H, s, H_3_13) ppm; ^13^C NMR (75 MHz, CDCl_3_): *δ*_C_ = 171.2 (s, C15), 147.9 (s, C5), 142.0 (s, C8), 134.4 (s, C9′), 124.5 (s, C9), 123.0 (d, C10′), 107.5 (t, C14), 101.9 (s, C6′), 96.7 (s, C2′), 77.5 (s, C1′), 72.7 (s, C5′), 65.2 (t, C11), 60.0 (t, C15′), 53.3 (d, C6), 48.1 (d, C2), 39.4 (s, C1), 37.0 (t, C4), 30.8 (t, C3^a^), 29.7 (t, C7′^a^), 28.5 (t, C4′^b^), 28.3 (t, C11′^b^), 27.8 (t, C3′^b^), 27.7 (t, C8′^b^), 26.7 (t, C7), 26.5 (q, C12), 25.8 (q, C12′), 25.1 (t, C10), 22.3 (q, C13′), 20.7 (q, C14′), 14.7 (q, C13) ppm, ^a,b^ assignments are interchangeable; IR (KBr): ῡ_max_ = 3430, 2940, 2860, 1755, 1670, 1450, 1370, 1190, 1030, 995, 940 cm^−1^; DCI-MS (NH*_3_*) *m*/*z* 518 (C_30_H_48_NO_6_)^+^ [M + NH_4_]^+^, 500 (C_30_H_44_O_6_)^+^ [M]^+^, 408, 378, 244, 206, 174, 151, 134, 116. ^1^H and ^13^C NMR spectra, see the Supplementary Material.

#### 4.3.2. Saponaceolide F (**2**)

Colorless powder; [α]_D_^22^ + 32.9 (*c* 7.4 mg/mL, CH_2_Cl_2_); R_f_ = 0.32 (RP18 TLC; MeOH-H_2_O, 4:1); ^1^H NMR (300 MHz, CDCl_3_): *δ*_H_ = 6.97 (1 H, td, *J* = 7.0 and 2.0 Hz, H8), 5.16 (1 H, br t, *J* = 7.0 Hz, H10′), 5.05 (1 H, br d, *J* = 5.5 Hz, H10), 4.90 (1 H, br s, H14a), 4.59 (1 H, br s, H14b), 4.46 (1 H, dd, *J* = 10.0 and 6.0 Hz, H11a), 4.45 (1 H, br d, *J* = 13.0, H15′a), 4.28 (1 H, br d, *J* = 13.0, H15′b), 4.27 (1 H, dd, *J* = 10.0 and 2.0 Hz, H11b), 2.5–2.7 (3 H, m, H_2_7 and *H*O10), 2.31 (1 H, m, H4a), 2.16 (1 H, m, H4b), 1.95 (1 H, m, H6), 2.05–1.0 (14 H, overlapped m’s, H_2_3, H_2_3′, H_2_4′, H_2_7′, H_2_8′, H_2_11′, H2, HO2′), 1.30 (3 H, s, H_3_12′), 1.23 (3 H, s, H_3_13′), 1.09 (3 H, s, H_3_14′), 1.07 (3 H, s, H_3_12), 0.63 (3 H, s, H_3_13) ppm; ^13^C NMR (75 MHz, CDCl_3_): *δ*_C_ = 169.9 (s, C15), 149.2 (s, C8), 147.9 (s, C5), 134.7 (s, C9′), 128.0 (s, C9), 123.0 (d, C10′), 108.3 (t, C14), 102.0 (s, C6′), 96.8 (s, C2′), 77.6 (s, C1′), 72.8 (s, C5′), 74.2 (t, C11), 66.3 (d, C10), 60.2 (t, C15′), 53.6 (d, C6), 48.3 (d, C2), 39.6 (s, C1), 36.9 (t, C4), 30.9 (t, C3^a^), 29.8 (t, C7′^a^), 28.6 (t, C4′^b^), 28.4 (t, C11′^b^), 27.9 (t, C3′^b^), 27.8 (t, C8′^b^), 26.8 (q, C12), 26.1 (t, C7), 25.9 (q, C12′), 22.5 (q, C13′), 20.8 (q, C14′), 15.1 (q, C13) ppm, ^a,b^ assignments are interchangeable; IR (KBr): ῡ_max_ = 3430, 2930, 2860, 1740, 1670, 1455, 1370, 1187, 1025, 990, 936 cm^−1^; DCI-MS (NH*_3_*) *m*/*z* 534 (C_30_H_48_NO_7_)^+^ [M + NH_4_]^+^, 516 (C_30_H_44_O_7_)^+^ [M]^+^, 408, 391, 373, 339, 221, 174, 163, 146, 128. ^1^H and ^13^C NMR spectra, see the Supplementary Material.

#### 4.3.3. Tricholidic acid (**5**)

Colorless solid; mp 208–212 °C (dec.); [α]_D_^22^—128.1 (*c* 3.6 mg/mL, CH_2_Cl_2_); [Δε]_225_^22^—2.08 (*c* 0.015, mg/mL, MeOH); R_f_ 0.64 (silica gel TLC; CH_2_Cl_2_-Me_2_CO, 6:1); ^1^H NMR and ^13^C NMR spectra, see Table 1; IR (KBr): ῡ_max_ = 3800–2800 (broad, medium-intensity band), 2930, 2860, 1750, 1730–1700 (broad band), 1690, 1625, 1455, 1250, 1040, 910 cm^−1^; DCI-MS (NH_3_) *m*/*z* 558 (C_32_H_48_NO_7_)^+^ [M + NH_4_]^+^, 541, 514, 497, 133, 116. ^1^H and ^13^C NMR spectra, see the Supplementary Material.

#### 4.3.4. Tricholidic acid B (**6**)

Colorless powder; [α]_D_^22^—102.3 (*c* 7.7 mg/mL, CH_2_Cl_2_); [Δε]_225_^22^—2.21 (*c* 0.02, mg/mL, MeOH); R_f_ 0.41 (RP18 TLC; MeOH-H_2_O, 6:1); ^1^H NMR and ^13^C NMR spectra, see Table 1; IR (KBr): ῡ_max_ = 3700–2700 (broad strong band), 2930, 1775, 1730–1680 (broad band), 1620, 1450, 1380, 1250, 1185, 1040, 1015, 965, 915 cm^−1^; DCI-MS (NH_3_) *m*/*z* 574 (C_32_H_48_NO_8_)^+^ [M+NH_4_]^+^, 556 (C_32_H_44_O_8_)^+^ [M]^+^, 539, 530, 512, 495, 296, 200, 133, 116. ^1^H and ^13^C NMR spectra, see the Supplementary Material.

#### 4.3.5. Tricholidic acid C (**7**)

Sticky oil; negative signs of optical rotation at 589 nm and CD at about 220 nm; R_f_ 0.74 (silica gel TLC; CH_2_Cl_2_-Me_2_CO, 8:1); ^1^H NMR and ^13^C NMR spectra, see Table 1; IR (KBr): ῡ_max_ = 3700–2700 (broad, medium intensity band), 2930, 2860, 1770, 1730, 1700, 1460, 1375, 1250, 1180, 1065, 1040, 1030, 975 cm^−1^; DCI-MS (NH_3_) *m*/*z* 544 (C_32_H_50_NO_6_)^+^ [M + NH_4_]^+^, 527, 500, 476, 274, 217, 200, 134. ^1^H and ^13^C NMR spectra, see the Supplementary Material.

#### 4.3.6. Tricholomenyn C (**8**)

Pale yellow oil; R_f_ 0.66 (RP18 TLC; MeOH-H_2_O, 6:1); ^1^H NMR (300 MHz) and ^13^C NMR (75 MHz) data (CDCl_3_) identical with those reported in the literature [24] and with an authentic sample.

### 4.4. Cytotoxicity (MTS) Assays

The MTS assay protocol is based on the reduction of the MTS [3-(4,5-dimethylthiazol-2-yl)-5-(3-carboxymethoxyphenyl)-2-(4-sulfophenyl)-2H-tetrazolium] yellow tetrazolium compound by NAD(P)H-dependent dehydrogenase enzymes in metabolically active cells to a purple formazan salt that is soluble in cell culture media [34]. The formazan salt has a maximum absorbance in the UV spectrum at 490 nm. The measure of the absorbance can be directly related to the number of viable (living) cells.

Five human cancer cell lines, myeloid leukemia HL-60, lung cancer A-549, hepatocellular cancer HepG2, renal cancer Caki-1, and breast cancer MCF-7, obtained from ATCC, were used in the MTS test. The cells were cultured in RPMI-1640 or DMEM/F-12 medium (Euroclone S.p.A, Milan, Italy) supplemented with 10% fetal bovine serum (FBS), 100 μg/mL penicillin/streptomycin and 2 mM L-glutamine (Life Technologies, Milan, Italy) at 37 °C under a humidified atmosphere added of 5% CO_2_, changing the liquid growth medium whenever needed. When a cell culture reached 75% confluence, a small amount of trypsin was added to the medium to separate the cells from the flask; after 3 min of incubation at 37 °C, 1 mL of FBS was added to stop the action of trypsin and to avoid degradation of cell membrane. The cell-containing medium was then transferred into a cell strainer and centrifuged (ALC 4232 Centrifuge) at 1000 rpm for 10 min. The resulting pellet was resuspended in the growth medium (1 mL), and the cells were separated using an automatic pipette and counted using a counting chamber and trypan blue as dye. Separated cells were seeded into each well of 96-well flat-bottom microplates (Cellstar, Greiner bio-one, Kremsmunster, Austria) at a density of 3 × 10^5^ cells/mL of growth medium in each well. After 2 h of incubation the medium was replaced with 100 µL of test medium (RPMI 1640, added with 0.005% L-glutamine, penicillin, and streptomycin), and the microplates were left in the incubator for 24 h. For the IC_50_ determination, six concentrations ranging from 0.1 to 100 µM in DMSO were prepared for each sample, including the cisplatin used as the cytotoxic reference compound. The medium in the wells was replaced with a solution (100 µL) of increasing sample concentration. Three replicates were performed for each sample dilution. The microplates were then incubated for 24 h; subsequently, the sample containing medium was replaced with fresh test medium (100 µL) and 20 µL of MTS tetrazolium reagent (CellTiter 96^®^—AQueous One Solution Cell Proliferation Assay, Promega Italia S.R.L., Milan, Italy). After 2 h incubation the absorbance at 490 nm was measured using a plate reader (BioRAD Model 550 Microplaate Reader). After each sample treatment, cell viability was detected, and a cell growth curve (mean absorbance vs. sample concentration) was graphed. IC_50_ values, expressed in μM (Table 2), were calculated by probit analysis (*p* < 0.05, χ^2^ test).

## 5. Conclusions

The first chemical analysis of the mushroom *Tricholoma ustaloides* led to the isolation and structure elucidation of two novel lanostane triterpenoids, named tricholidic acids B (**6**) and C (**7**). In addition, known saponaceolides J (**1**) and F (**2**), tricholidic acid (**5**), and tricholomenyn C (**8**), together with triglycerides, a mixture of free fatty acids, and five unidentified compounds A1–A5, were isolated from an EtOAc extract of the fruiting bodies. This is the second isolation of metabolites **1**, **2**, **5**, and **8** from nature. Moreover, saponaceolides J (**1**) and F (**2**) showed high cytotoxicity (IC_50_ values ≤ 10 μM) in an MTS assay against a panel of human cancer cells.

Our findings have confirmed that *Tricholoma* species are rich sources of novel compounds, most of which have unique chemical structures and interesting bioactivities. Therefore, we will further investigate the chemical contents of *Tricholoma* species that grow in the wild in Italy. Regarding the metabolites of *T. ustaloides*, we aim to establish the structures of the still unidentified compounds, A1–A5, that have been isolated in this study.

## Figures and Tables

**Table 1:** ^1^H NMR (300 MHz, CDCl_3_, *δ*_H_ in ppm, *J* in Hz) and ^13^C NMR (75 MHz, CDCl_3_, *δ*_C_ in ppm) spectroscopic data of. compounds **5**–**7**.

Position	5		6		7	
H/C	*δ*_C_ ^a^	*δ*_H_ ^b,c^	*δ*_C_ ^a^	*δ*_H_ ^b,c^	*δ*_C_ ^a^	*δ*_H_ ^b,c^
1	30.9 ^d^ t	1.52 m	30.5 t	1.53 m	30.7 ^d^ t	1.55 m
2	23.1 t	1.72 m	23.1 t	1.71 m	23.2 t	1.65–1.78 m
3	77.4 d	4.67 t (2.5)	77.7 d	4.66 t (2.5)	77.6 d	4.67 t (2.5)
4	36.8 ^e^ s	--	36.8 ^d^ s	--	36.8^e^ s	--
5	44.8 d	1.55 m	44.7 d	1.55 m	45.0 d	1.57 m
6	17.7 t	1.47 m 1.68 m	17.8 t	1.45 m 1.66 m	17.8 t	1.55 m 1.65 m
7	29.5 t	^g^	29.7 t	^g^	29.7 t	^g^
8	127.3 s	--	127.0 s	--	128.1 s	--
9	142.0 s	--	141.6 s	--	142.3 s	--
10	37.6 ^e^ s	--	37.6 ^d^ s	--	37.7 ^e^ s	--
11	27.7 t	2.08 m	27.6 t	2.05 m	27.8 t	2.10 m
12	30.7 ^d^ t	2.21 m	30.5 t	2.19 m	31.0 ^d^ t	1.75–2.0 m
13	46.7 s	--	44.8 s	--	46.9 s	--
14	64.2 s	--	64.3 s	--	64.4 s	--
15	36.1 t	1.97 dd (13.9, 4.0) 2.73 dd (13.9, 7.5)	35.2 t	1.93 dd (13.9, 4.3) 2.76 dd (13.9, 7.6)	36.3 t	2.00 m 2.68 m
16	83.7 d	5.15 ddd (7.5, 7.0, 4.1)	83.8 d	5.42 ddd (7.5, 6.3, 4.2)	83.2 d	5.06 m
17	48.9 d	2.87 dd (8.7, 6.7)	58.3 d	2.72 d (6.3)	49.4 d	2.70 m
18	18.5 ^f^ q	0.92 s	18.6 ^e^ q	0.92 s	18.2 ^f^ q	0.92 s
19	19.2 ^f^ q	0.94 s	19.2 ^e^ q	0.95 s	19.1 ^f^ q	0.92 s
20	38.0 d	3.46 td (8.9, 4.0)	76.6 s	--	42.9 d	2.70 m
21	178.6 s	--	179.3 s	--	178.7 s	--
22	40.4 t	2.82 dd (18.6, 9.1) 3.15 dd (18.6, 4.0)	43.6 t	2.86 d (18.0) 3.21 d (18.0)	27.1 t	2.0–2.20 m
23	196.9 s	--	200.5 s	--	22.0 t	2.2–2.35 m
24	122.9 d	6.12 sept (1.2)	123.8 d	6.12 m (1.2)	123.2 d	5.10 m
25	157.2 s	--	158.7 s	--	133.2 s	--
26	27.5 q	1.93 d (1.2)	28.0 q	1.95 d (1.2)	17.8 q	1.61 br s
27	21.0 q	2.16 d (1.2)	21.3 q	2.19 d (1.2)	25.7 q	1.70 br s
28	27.7 q	0.87 s	27.5 q	0.88 s	27.5 q	0.86 s
29	21.8 q	1.04 s	21.8 q	1.04 s	21.8 q	1.04 s
30	177.6 s	--	175.6 s	--	177.2 s	--
31	170.7 s	--	170.7 s	--	170.6 s	--
32	21.3 q	2.05 s	21.3 q	2.05 s	21.2 q	2.08 s

^a^ Carbons multiplicities were determined by DEPT experiments; ^b^ the center of the signal is reported as the chemical shift of a multiplet; ^c^ the numbers in parentheses are the coupling constants; ^d,e,f^ assignments in each vertical column can be interchanged, ^g^ multiplet overlapped by other signals.

**Table 2:** Cytotoxicities of compounds **1**–**7** and cisplatin against five human cancer cell lines.

IC_50_ (μM)					
Compound/Cell Line	HL-60	A-549	Hep-G2	Caki-1	MCF-7
saponaceolide J (**1**)	1.5 ± 0.10	8.5 ± 0.20	5.0 ± 0.19	10.3 ± 0.25	6.4 ± 0.18
saponaceolide F (**2**)	0.3 ± 0.05	0.9 ± 0.10	1.8 ± 0.11	4.2 ± 0.17	4.5 ± 0.15
saponaceolide B (**3**)	1.1 ± 0.12	13.8 ± 0.35	6.3 ± 0.19	>20	2.1 ± 0.11
saponaceolide A (**4**)	0.3 ± 0.09	1.9 ± 0.13	3.0 ± 0.18	12.5 ± 0.29	1.5 ± 0.10
tricholidic acid (**5**)	>25	n.d.	n.d.	n.d.	n.d.
tricholidic acid B (**6**)	>25	n.d.	n.d.	n.d.	n.d.
tricholidic acid C (**7**)	>25	n.d.	n.d.	n.d.	n.d.
cisplatin	n.d.	12.0 ± 0.30	13.5 ± 0.32	16.0 ± 0.40	17.6 ± 0.39

n.d. = the activity value was not determined.

## Data Availability

Raw data are available from one of the authors (G.V.).

## Supplementary Materials

^1^H and ^13^C NMR spectra of compounds **1**, **2**, **5**–**7** are available online at https://www.mdpi.com/article/10.3390/molecules28093864/s1.

## Sample Availability

Samples of the compounds are not available from the authors.
