# Supplementary material for: New Tricholidic Acid Triterpenoids from the Mushroom Tricholoma ustaloides Collected in an Italian Beech Wood
Source: Molecules. 2023 May 4;28(9):3864. doi: 10.3390/molecules28093864 (PMC10180436; doi:10.3390/molecules28093864)
Supplement: Supplementary file 1 [file molecules-28-03864-s001.zip › molecules-2297025-supplementary.pdf]

SUPPLEMENTARY MATERIALS

New tricholidic acid triterpenoids from the mushroom *Tricholoma ustaloides* collected in an Italian beech wood

Gianluca Gilardoni[1], Francesca Negri[2], Paola Vita Finzi[2], Faiq H. S. Hussain[3], Giovanni Vidari[2,3,*]

[1] Departamento de Química, Universidad Técnica Particular de Loja (UTPL), 1101608 Loja, Ecuador
[2] Dipartimento di Chimica, Università degli Studi di Pavia, Via Taramelli 10, 27100 Pavia, Italy
[3] Department of Medical Analysis, Faculty of Applied Science, Tishk International University, Erbil 44001, Kurdistan Region, Iraq
* Correspondence: vidari@unipv.it (G.V.)

Abstract: The secondary metabolites produced by *Tricholoma ustaloides* Romagn., a mushroom species belonging to the large *Tricholoma* genus (Basidiomycota, Tricholomataceae), are unknown. Therefore, encouraged by the interesting results obtained in our previous chemical analyses of a few *Tricholoma* species collected in Italian woods, we were aimed to investigate the secondary metabolites of *Trichooma ustaloides*. The chemical analysis involved the isolation and characterization of secondary metabolites through an extensive chromatographic study. The structures of isolated metabolites, including the absolute configuration, were established based on a detailed analysis of MS, NMR spectroscopic, optical rotation and circular dicroism data, and on comparison with those of related compounds reported in the literature. Two novel lanostane triterpenoids, named tricholidic acids B and C, together with triglycerides, a mixture of free fatty acids, five unidentified metabolites, and known rare saponaceolides F and J, tricholidic acid, and tricholomenyn C, were isolated from an EtOAc extract of fruiting bodies of *Tricholoma ustaloides*, that were collected in an Italian beech wood. This is the second example of isolation of tricholidic acid derivatives from a natural source. Saponaceolides F and J exibited high cytotoxicity (IC_{50} values ≤ 10 μM) against a panel of five human cancer cell lines. The toxicity against myeloid leukemia (HL-60), lung cancer (A-549), hepatocellular cancer (HepG2), renal cancer (Caki-1), and breast cancer (MCF-7) cells was higher than that shown by the very well-known cytotoxic drug cisplatin.

Keywords: *Tricholoma ustaloides*; Tricholomataceae (Basidiomycota); lanostane triterpenoids; tricholidic acid derivatives; saponaceolides; tricholomenyn; cytotoxicity.

^{1}H NMR spectrum of saponaceolide J (**1**)	S1A + S1B
^{13}C NMR spectrum of saponaceolide J (**1**)	S2A + S2B
^{1}H NMR spectrum of saponaceolide F (**2**)	S3
^{13}C NMR spectrum of saponaceolide F (**2**)	S4
^{1}H NMR spectrum of tricholidic acid (**5**)	S5
COSY spectrum of tricholidic acid (**5**)	S6
^{13}C NMR spectrum of tricholidic acid (**5**)	S7
DEPT (135°) of tricholoidic acid (**5**)	S8A + S8B
^{1}H NMR spectrum of tricholidic acid B (**6**)	S9

COSY spectrum of tricholodic acid B (**6**) S10

^{1}H NMR spectrum of tricholidic acid B methyl ester S11A + S11B

^{13}C NMR spectrum of tricholidic acid B (**6**) S12A + S12B

DEPT (135°) of tricholoidic acid B (**6**) S13

HSQC spectrum of tricholidic acid B (**6**) S14A – S14D

^{1}H NMR spectrum of tricholidic acid C **(7**) S15

COSY spectrum of tricholidic acid C (**7**) S16A + S16B

^{13}C NMR spectrum of tricholidic acd C (**7**) S17

DEPT (135°) of tricholoidic acid C (**7**) S18A – S18C

HSQC spectrum of tricholidic acid C (**7**) S19A – S19C

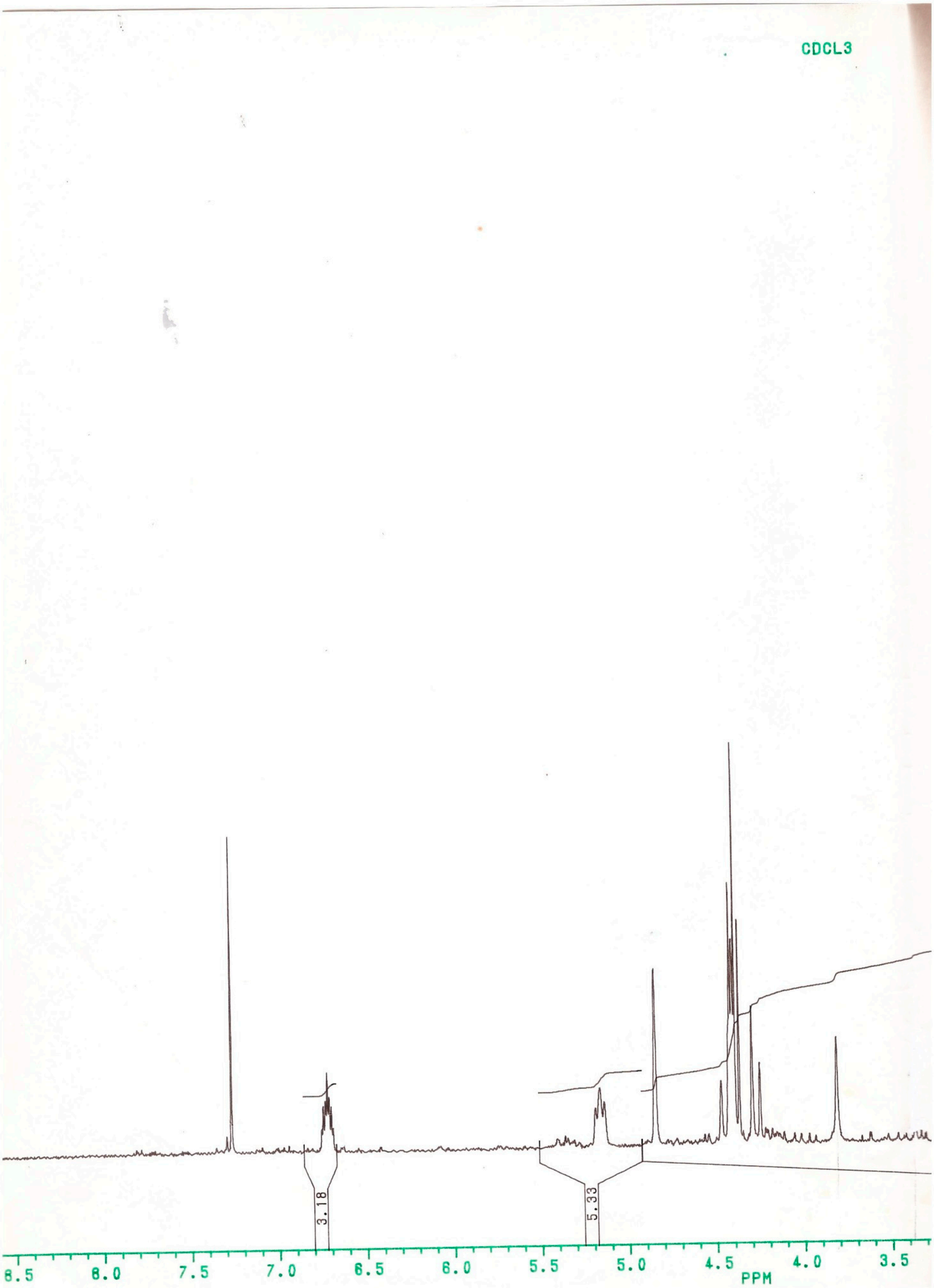

S1A

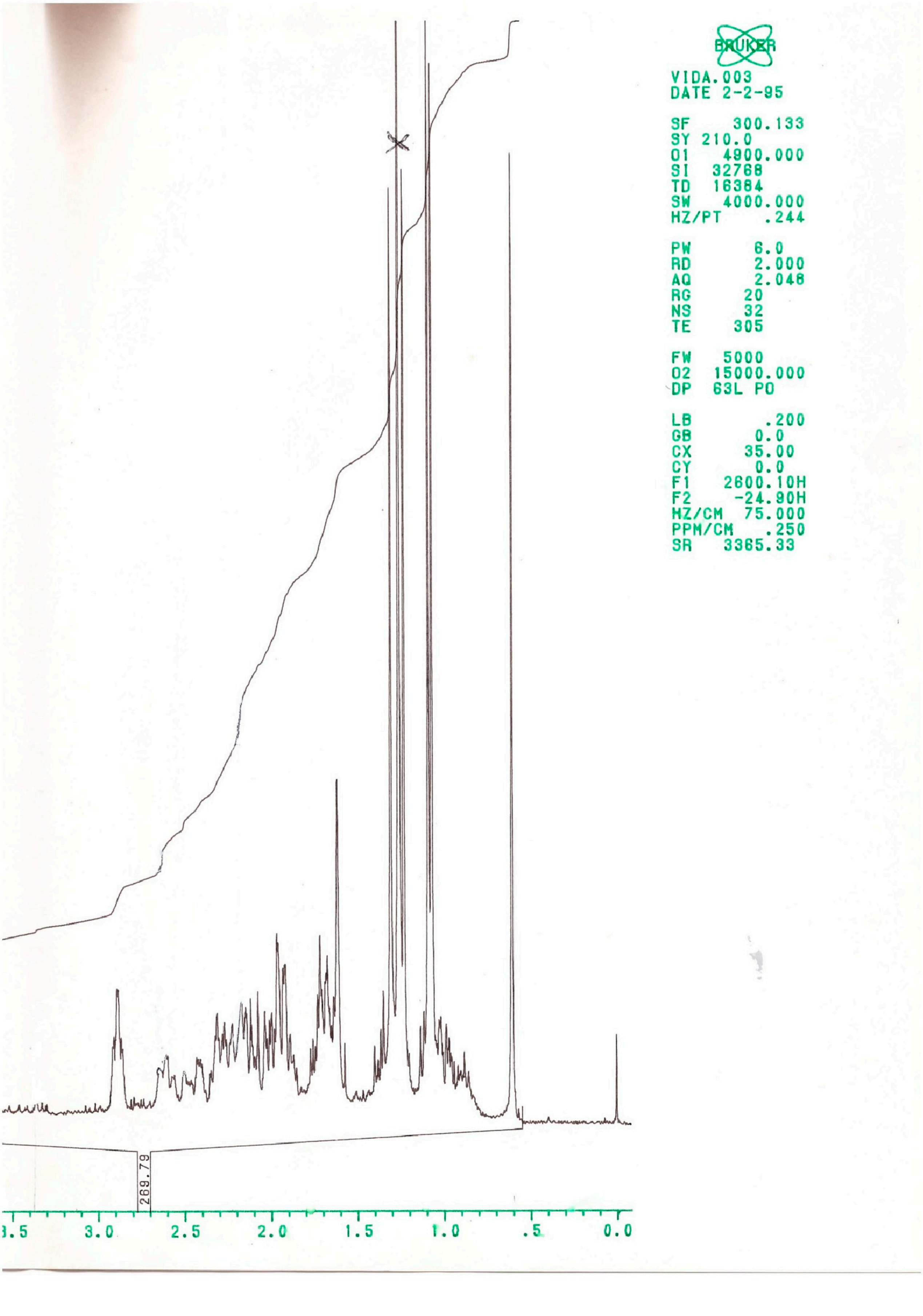
BRUKER
VIDA.003
DATE 2-2-95
SF 300.133
SY 210.0
O1 4900.000
SI 32768
TD 16384
SW 4000.000
HZ/PT .244
PW 6.0
RD 2.000
AQ 2.048
RG 20
NS 32
TE 305
FW 5000
O2 15000.000
DP 63L PO
LB .200
GB 0.0
CX 35.00
CY 0.0
F1 2600.10H
F2 -24.90H
HZ/CM 75.000
PPM/CM .250
SR 3365.33
269.79
3.5 3.0 2.5 2.0 1.5 1.0 .5 0.0

S1B

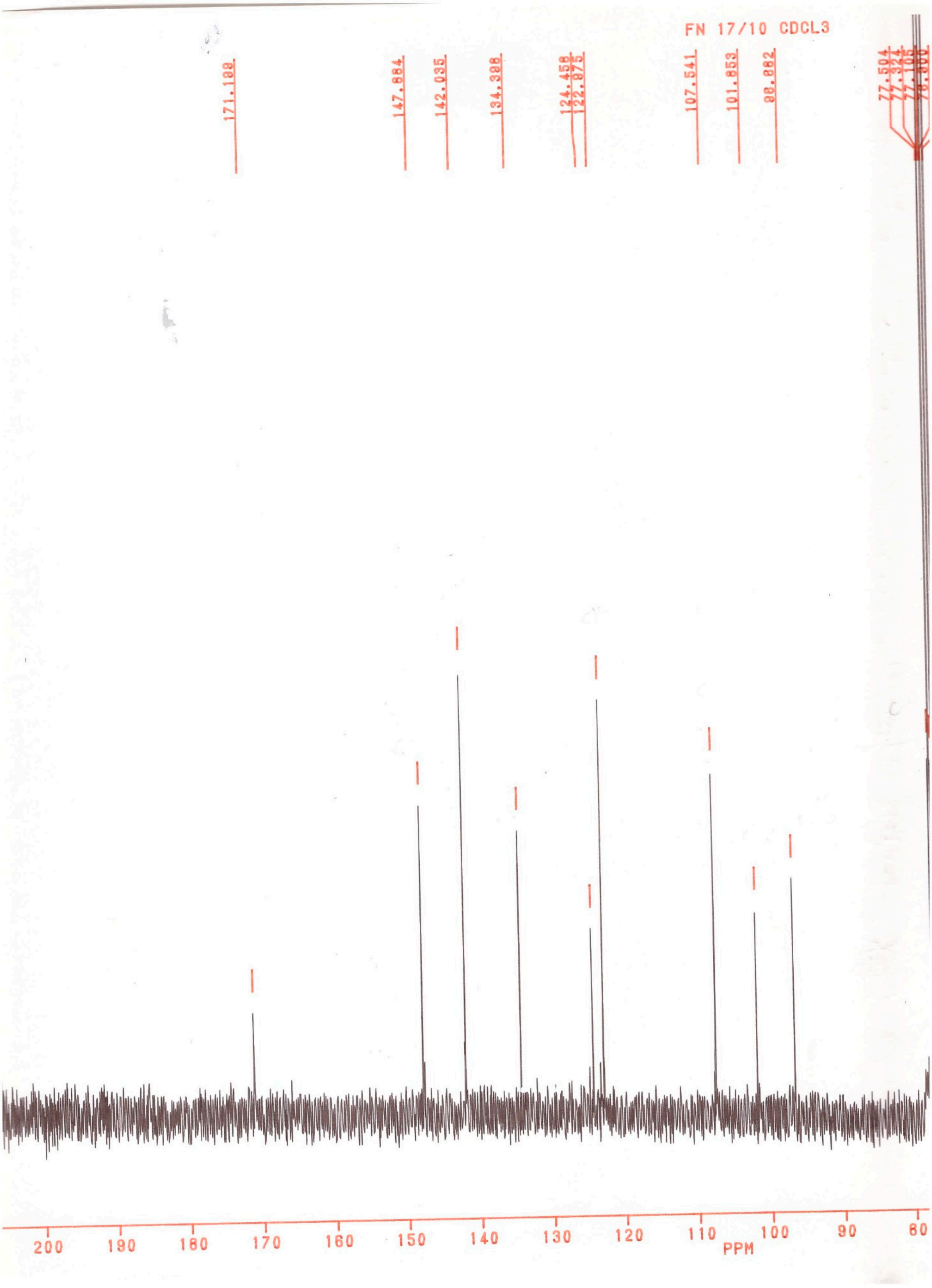
FN 17/10 CDCL3
171.199
147.884
142.035
134.388
124.458
122.875
107.541
101.853
98.882
PPM
200
190
180
170
160
150
140
130
120
110
100
90
80

S2A

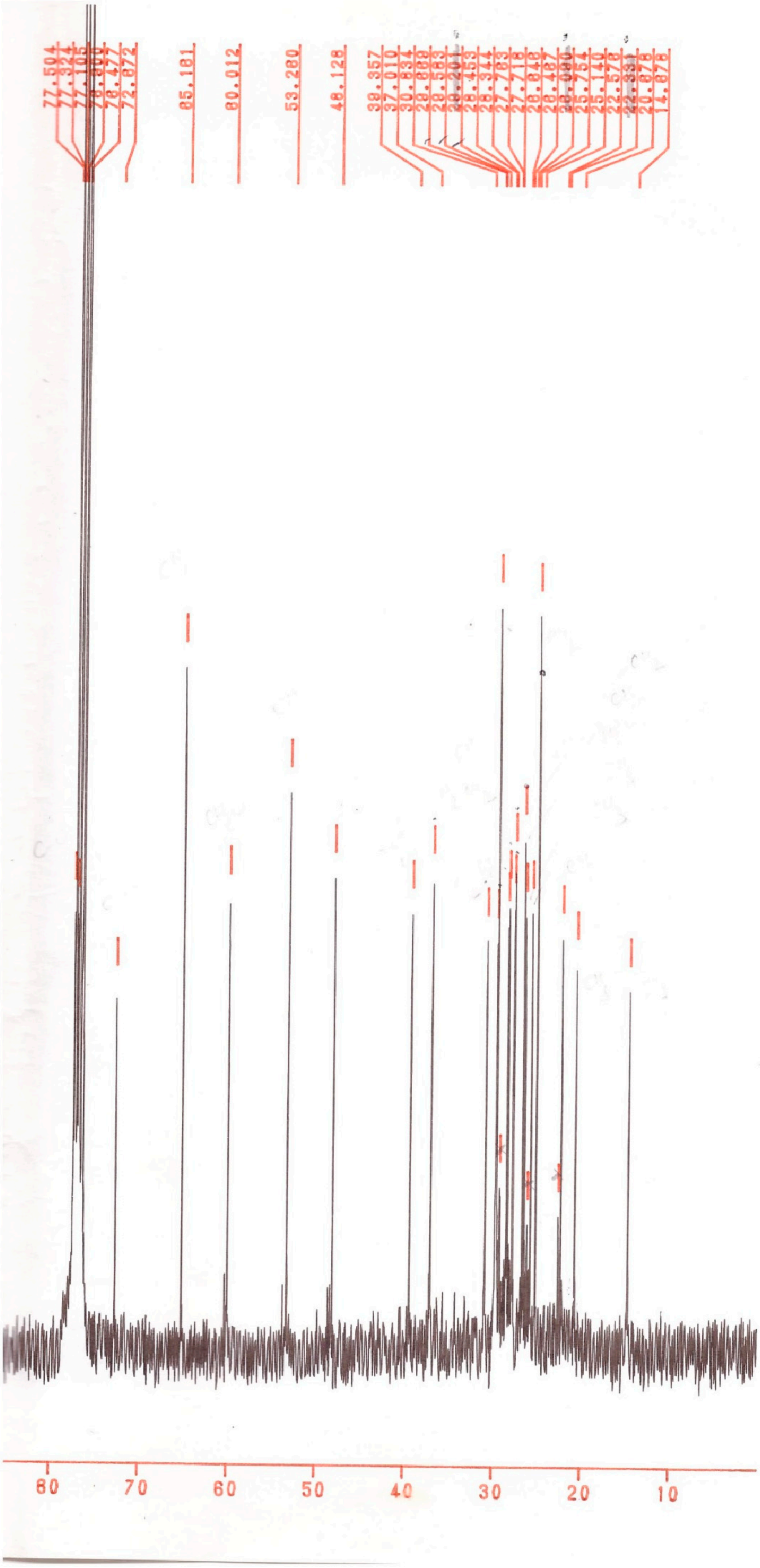
77.504
72.872
65.181
60.012
53.280
48.128
39.357
37.010
14.878
80
70
60
50
40
30
20
10

S2B

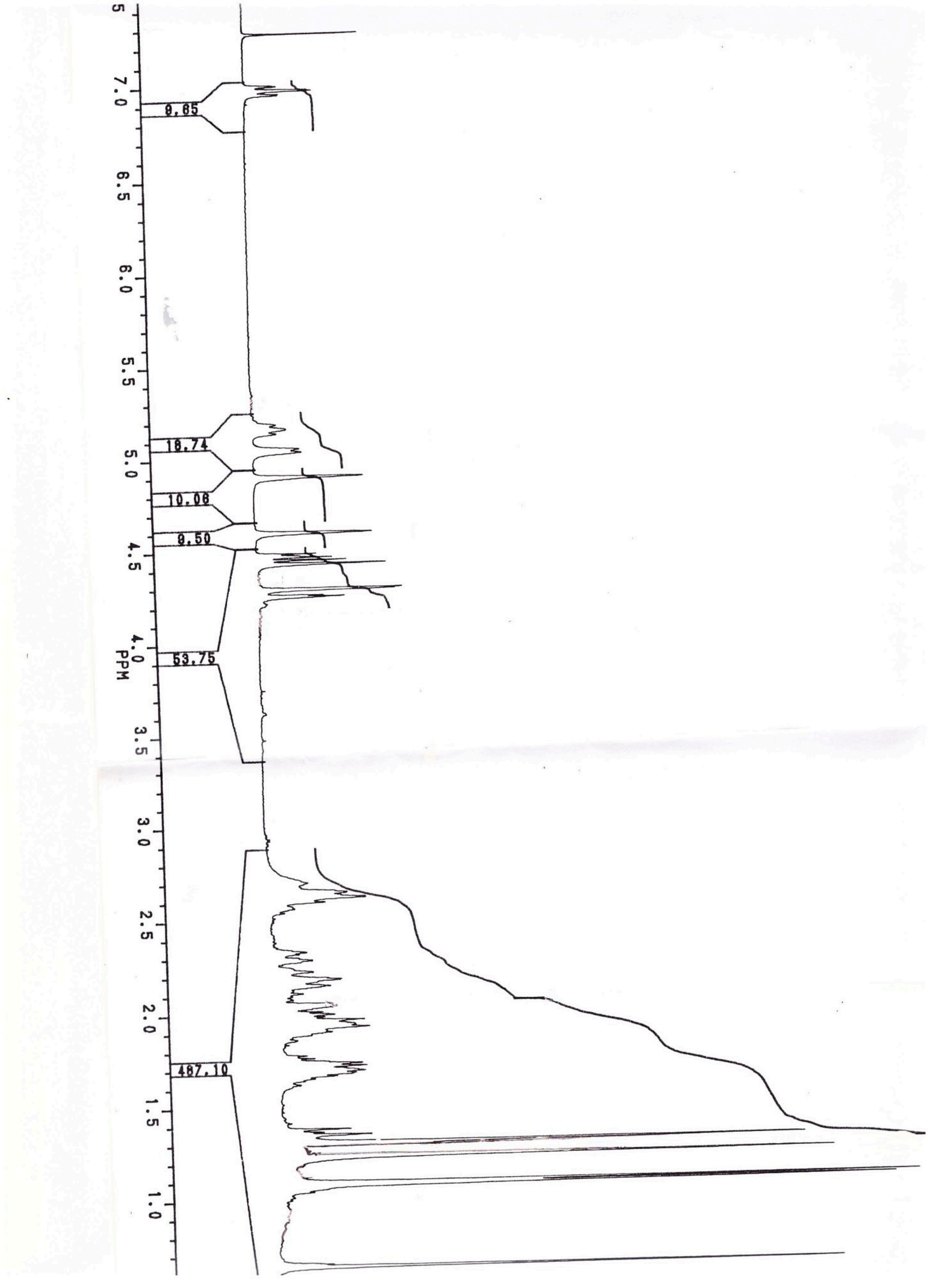
7.0
6.5
6.0
5.5
5.0
4.5
4.0
PPM
3.5
3.0
2.5
2.0
1.5
1.0
9.65
18.74
10.06
9.50
53.75
487.10

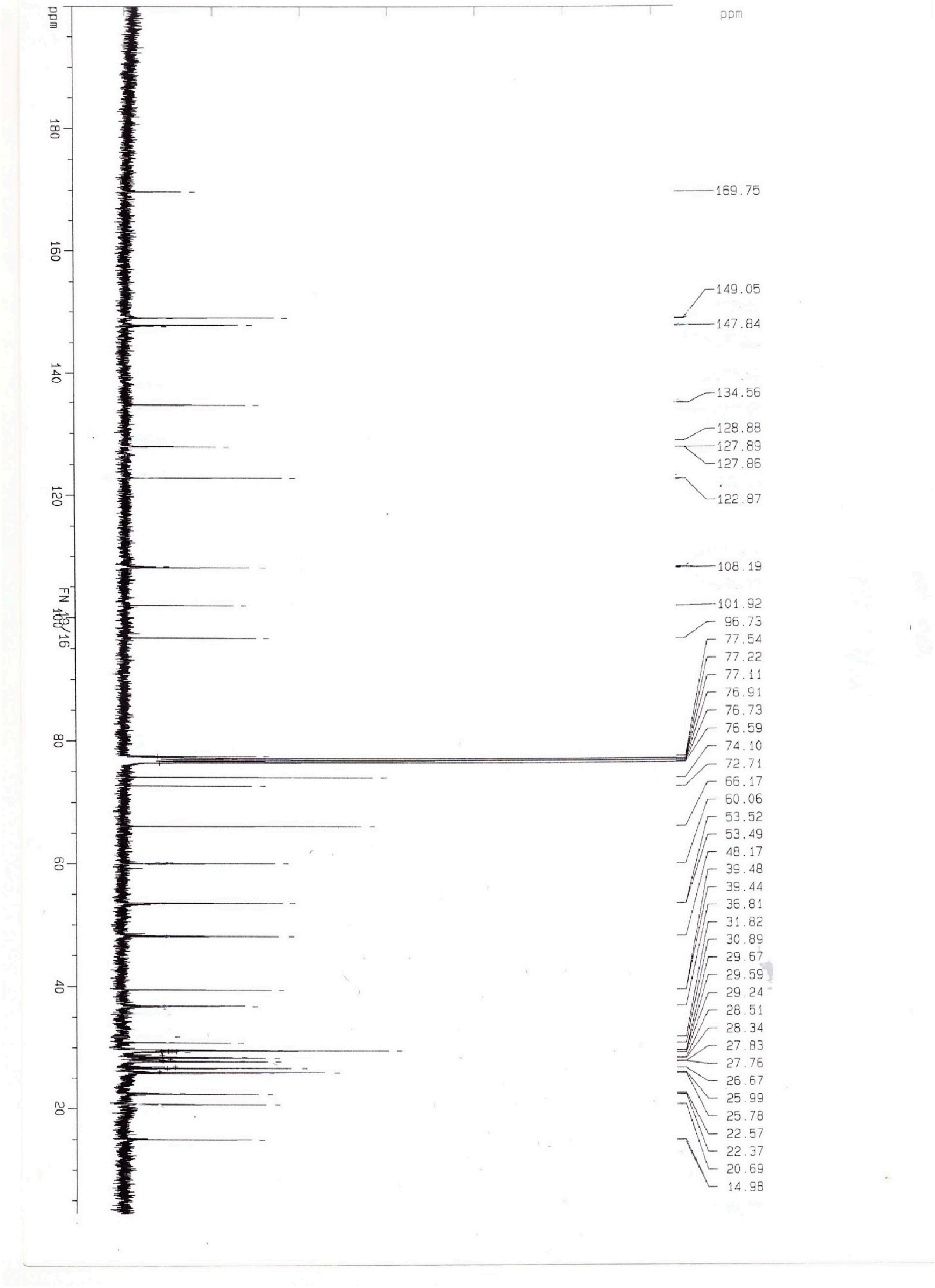
ppm
169.75
149.05
147.84
134.56
128.88
127.89
127.86
122.87
108.19
101.92
96.73
77.54
77.22
77.11
76.91
76.73
76.59
74.10
72.71
66.17
60.06
53.52
53.49
48.17
39.48
39.44
36.81
31.82
30.89
29.67
29.59
29.24
28.51
28.34
27.83
27.76
26.67
25.99
25.78
22.57
22.37
20.69
14.98

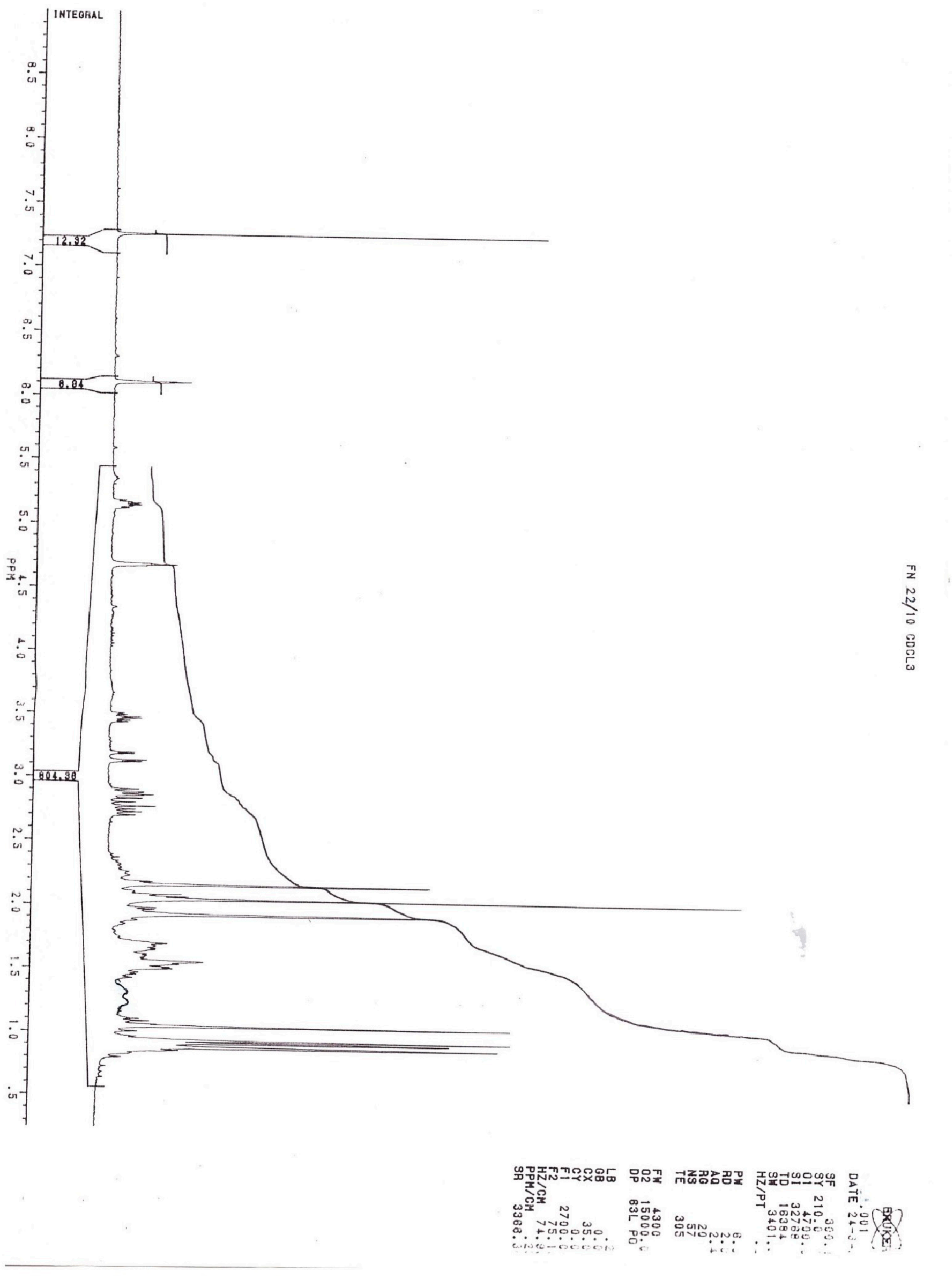
INTEGRAL
PPM
FN 22/10 CDCL3
12.92
6.04
804.98

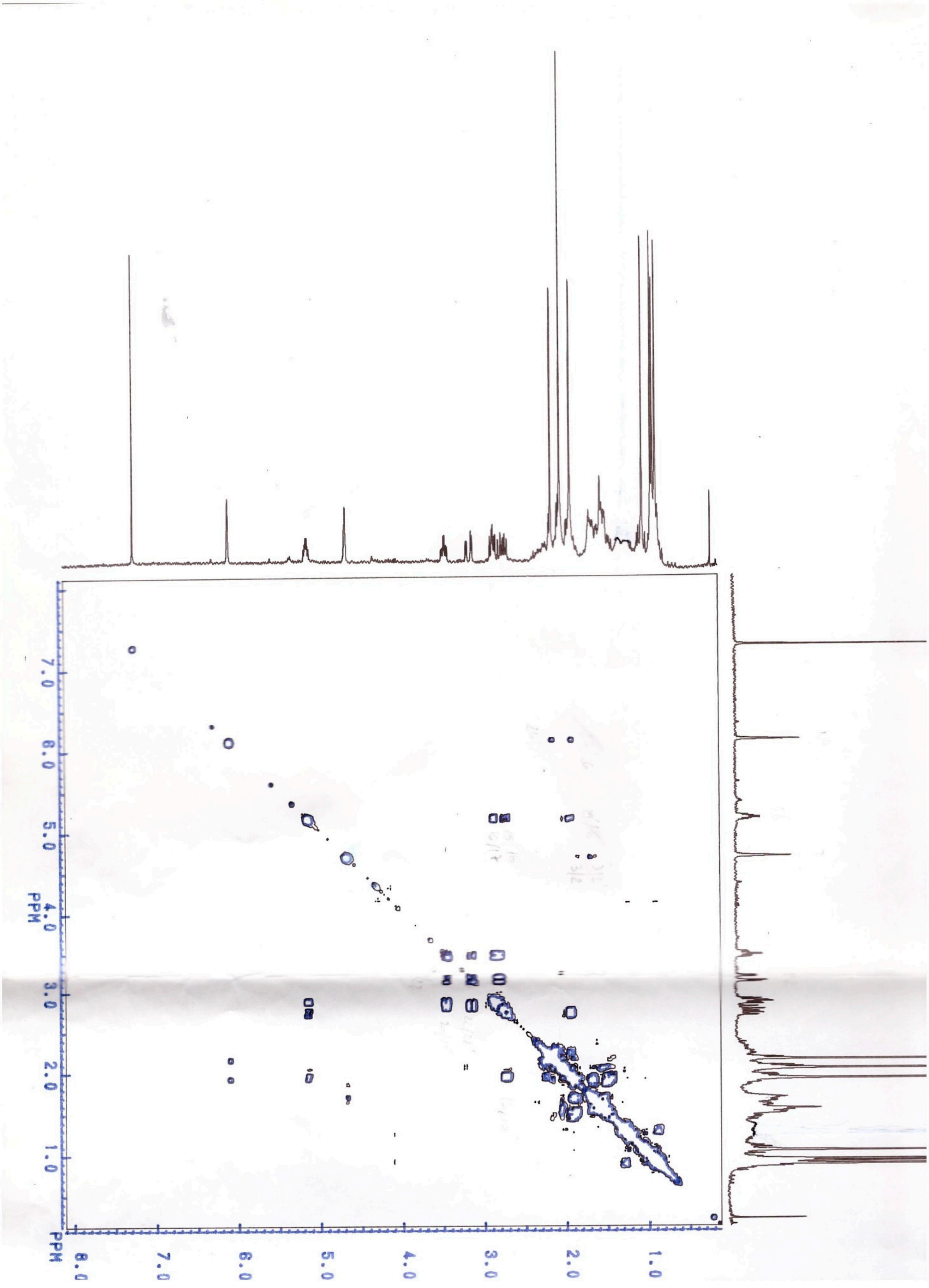
PPM
8.0
7.0
6.0
5.0
4.0
3.0
2.0
1.0

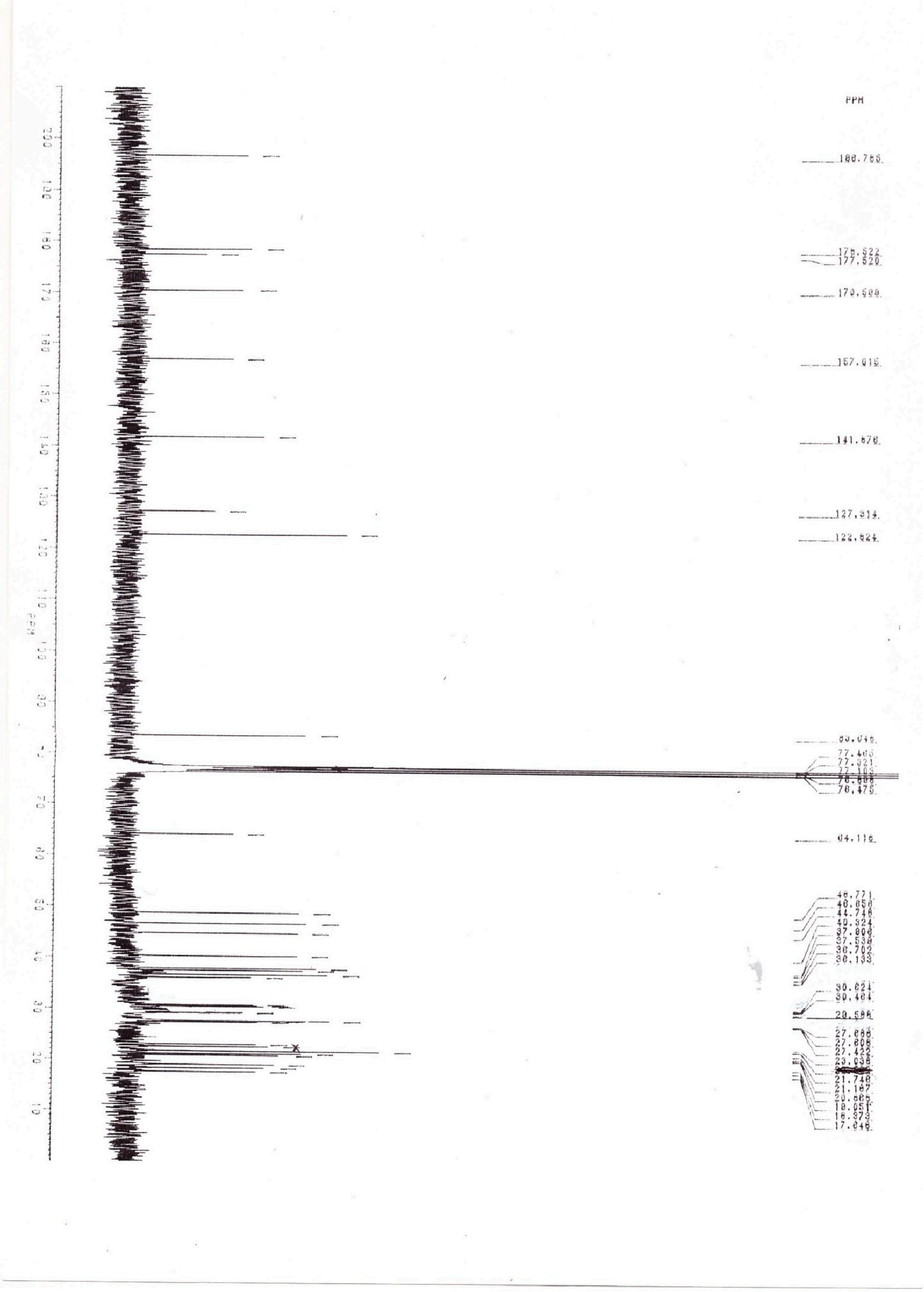

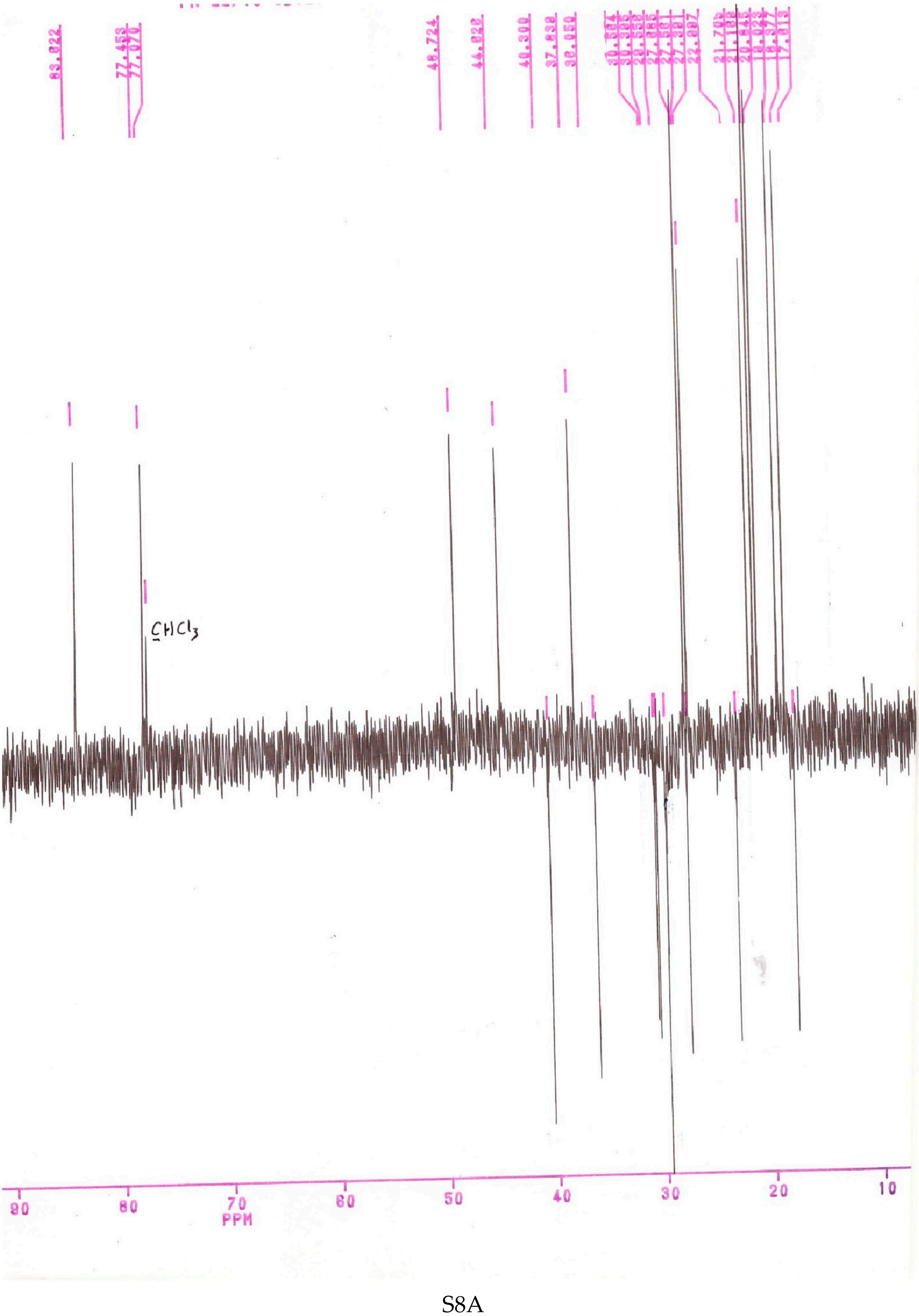

S8A

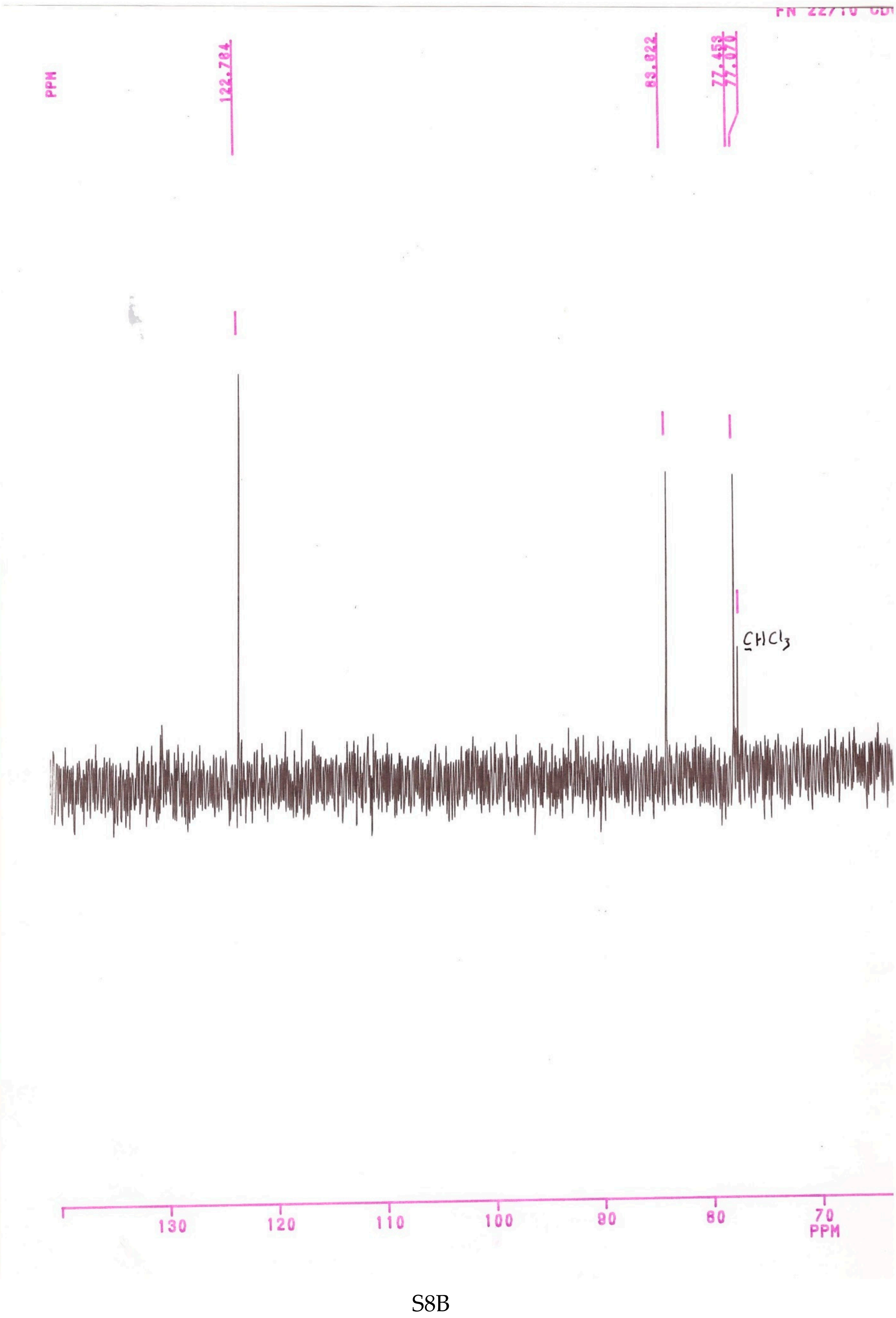
PPM
122.784
83.822
77.453
$CHCl_3$

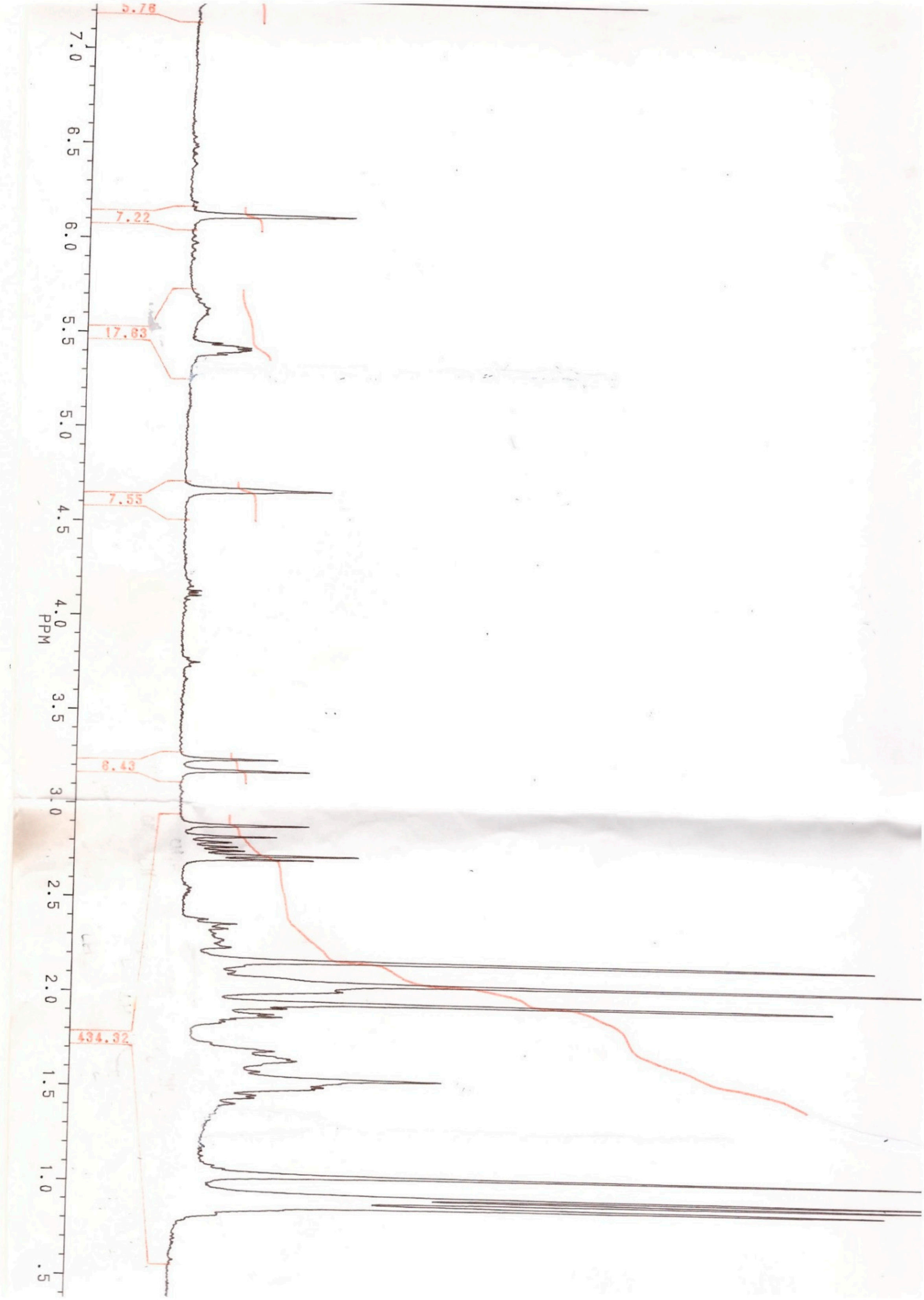

5.78
7.0
6.5
7.22
6.0
17.63
5.5
5.0
7.55
4.5
4.0
PPM
3.5
6.43
3.0
2.5
2.0
434.32
1.5
1.0
.5

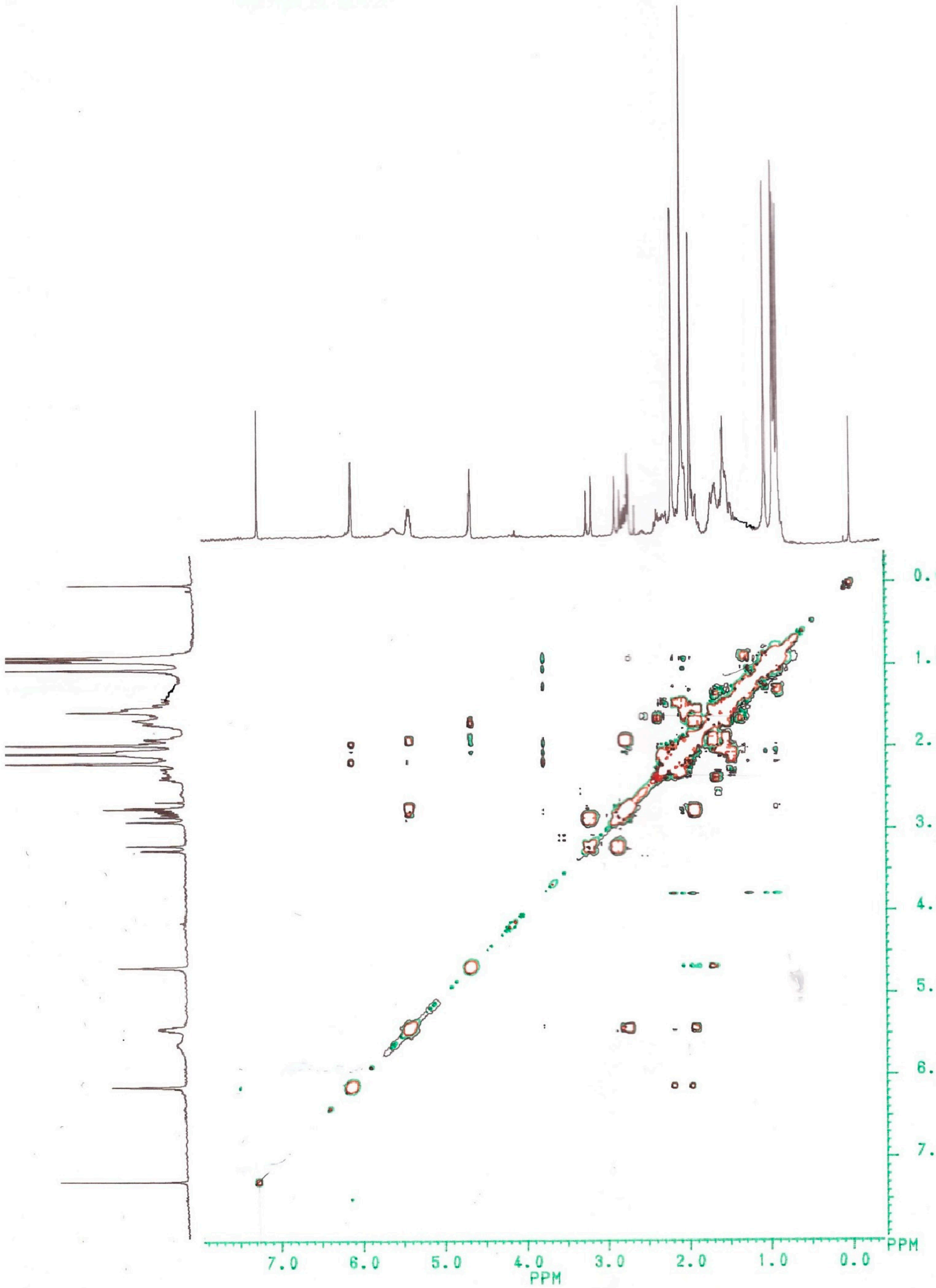
0.0
1.0
2.0
3.0
4.0
5.0
6.0
7.0
PPM
7.0 6.0 5.0 4.0 3.0 2.0 1.0 0.0
PPM

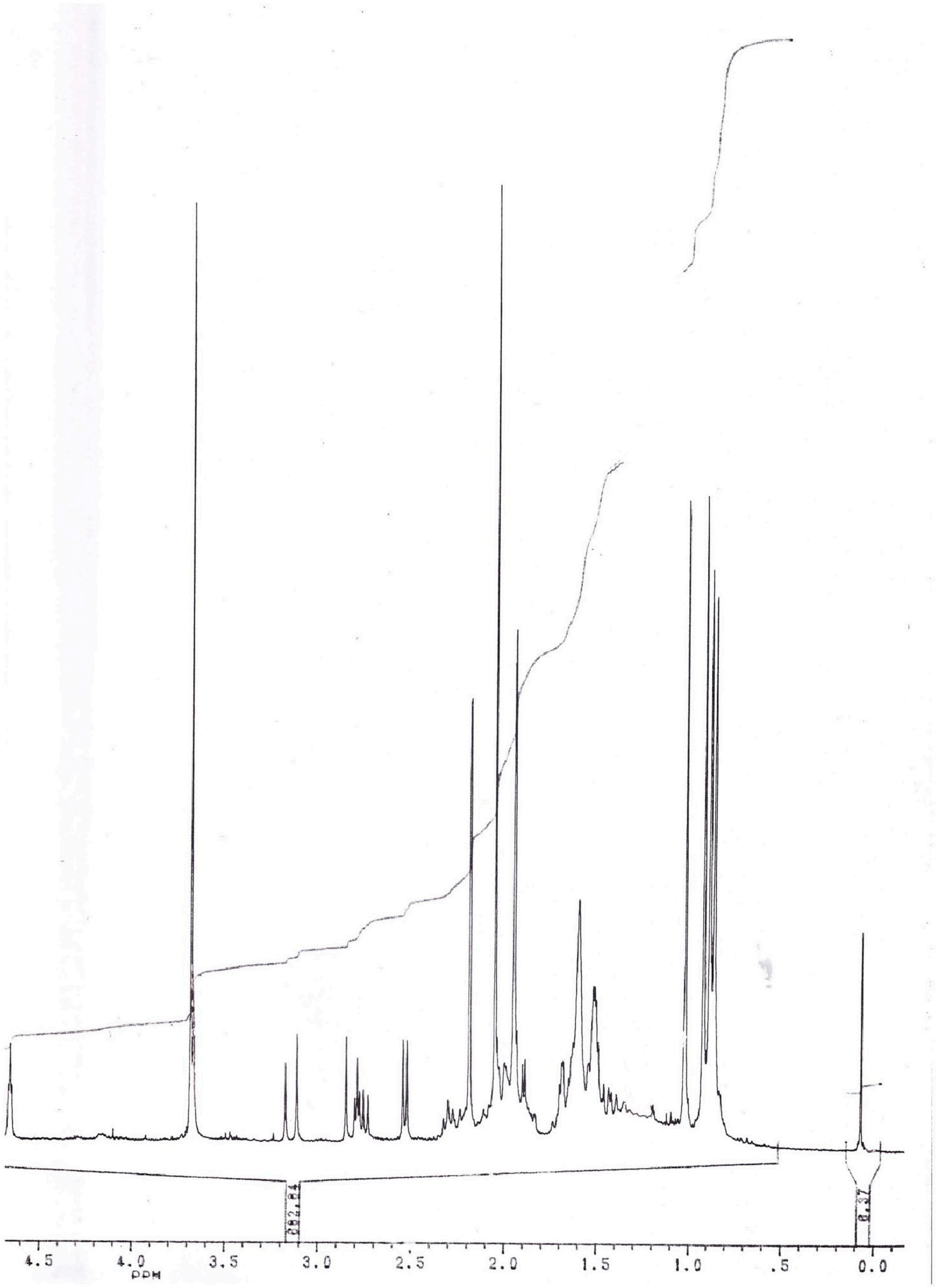

002.84
0.37
4.5
4.0
PPM
3.5
3.0
2.5
2.0
1.5
1.0
.5
0.0

S11A

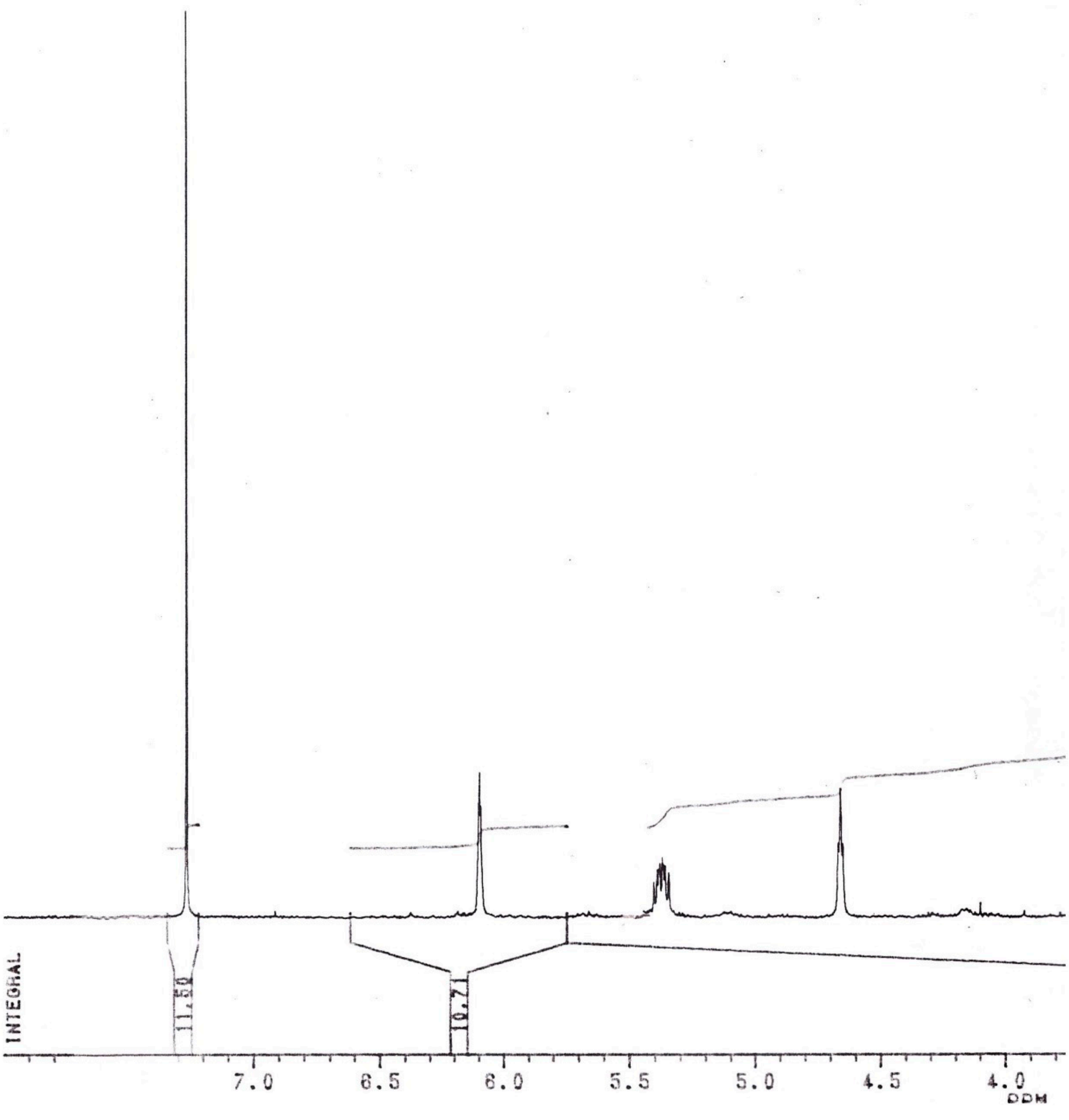

S11B

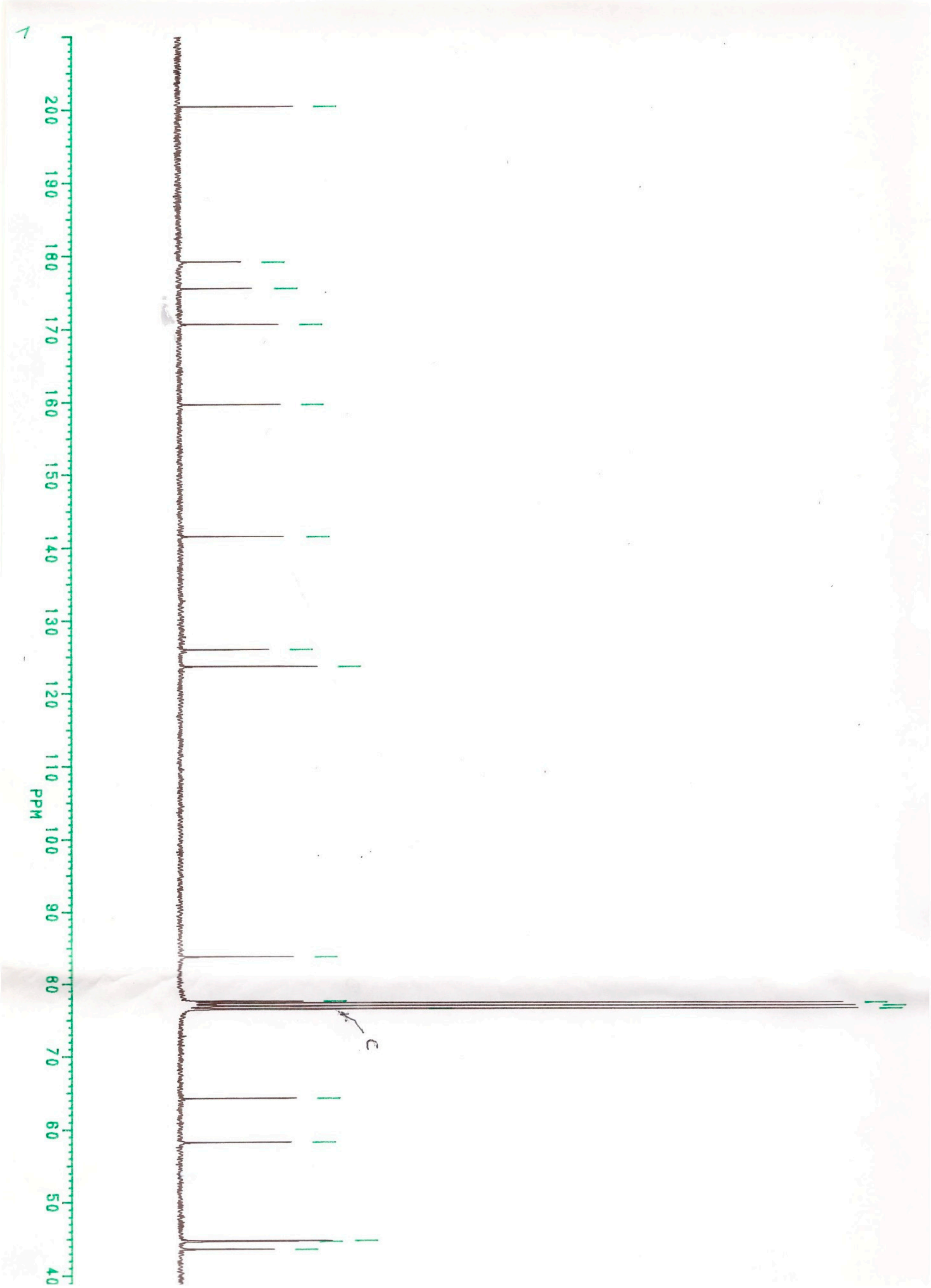

S12A

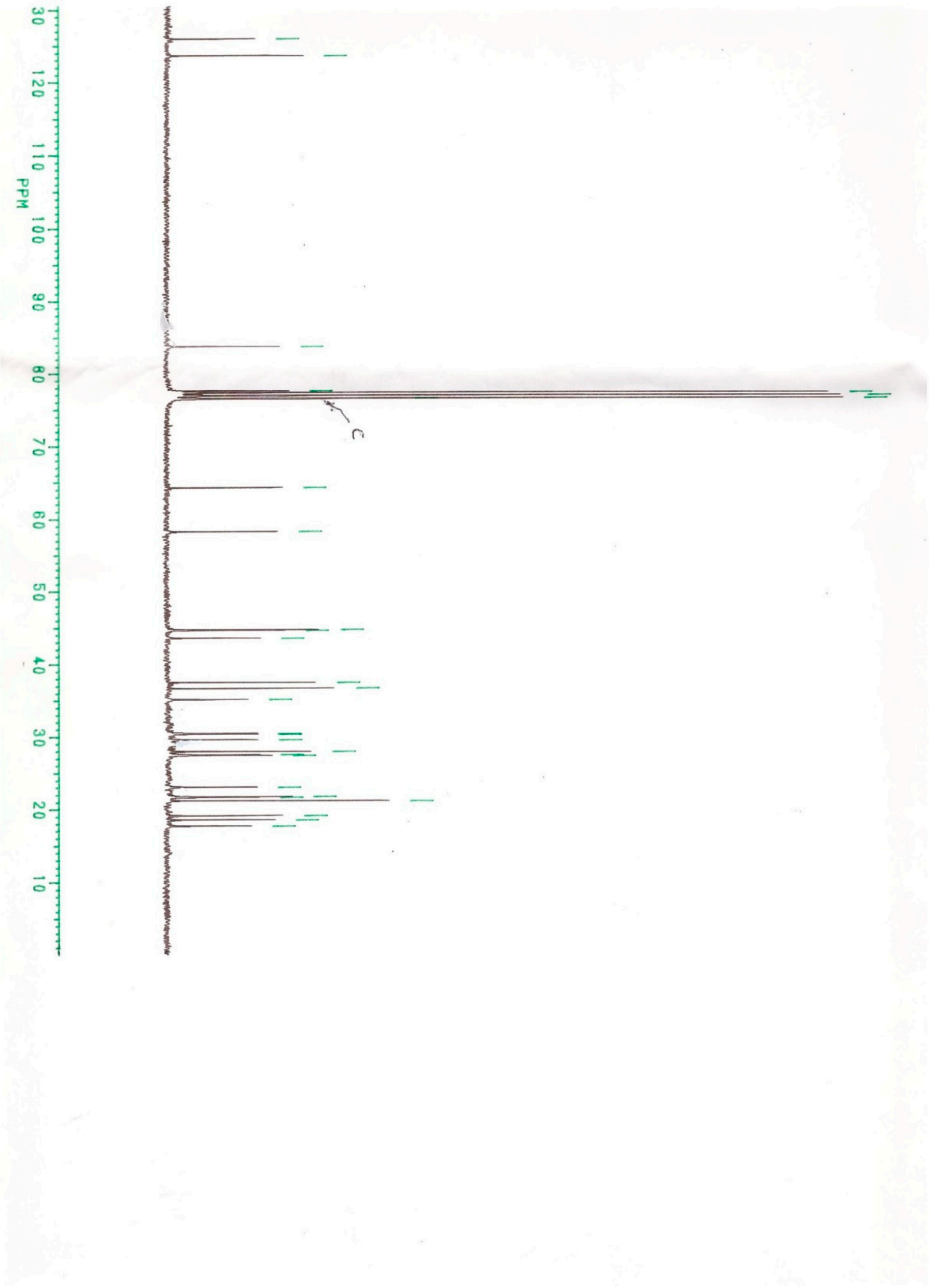

S12B

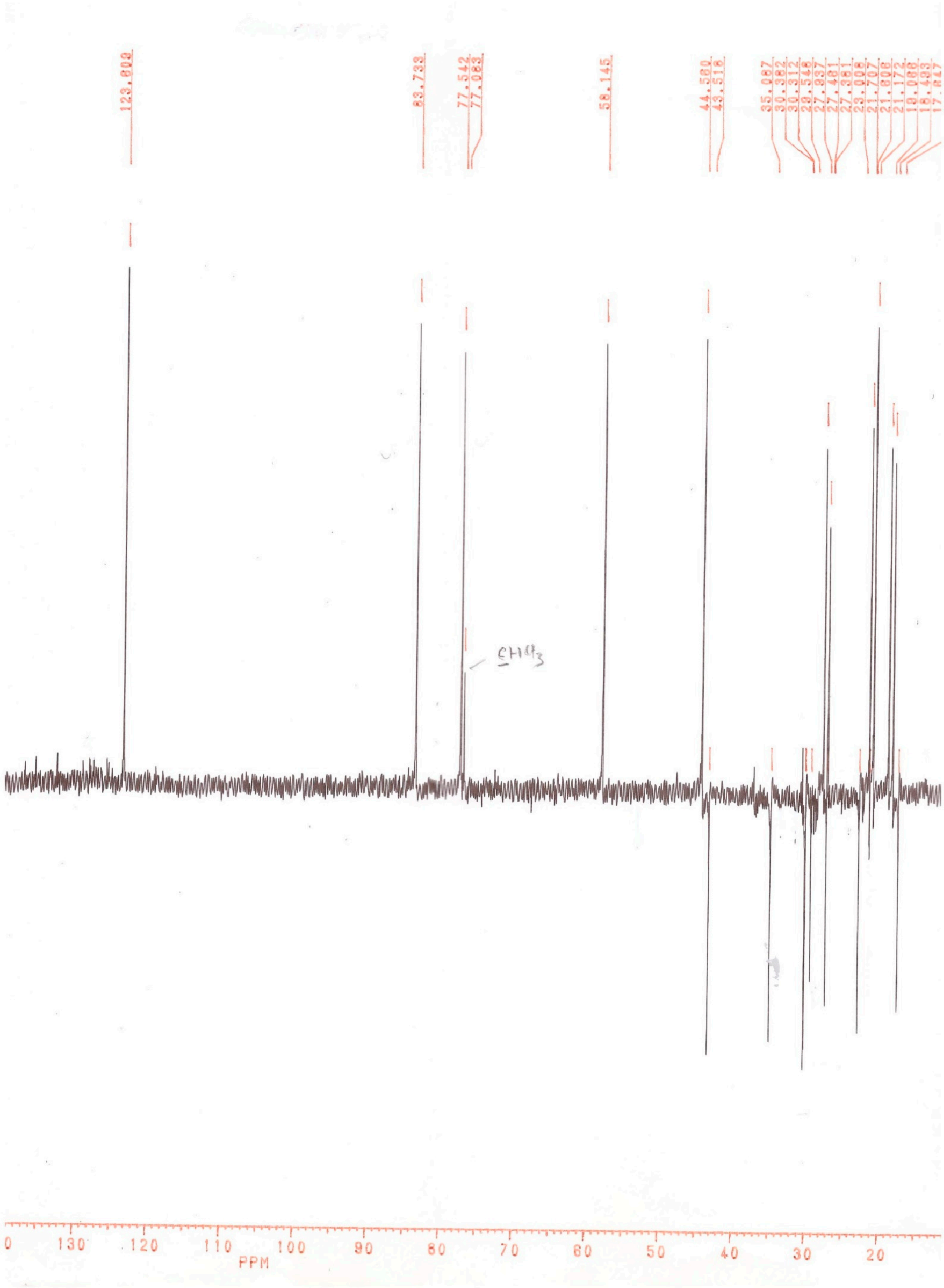

123.800
83.733
77.542
77.083
58.145
44.580
43.518
35.087
30.382
30.312
29.548
27.937
27.481
27.381
23.008
21.707
21.606
21.172
19.088
18.493
CHCl3
130
120
110
100
90
80
70
60
50
40
30
20
PPM

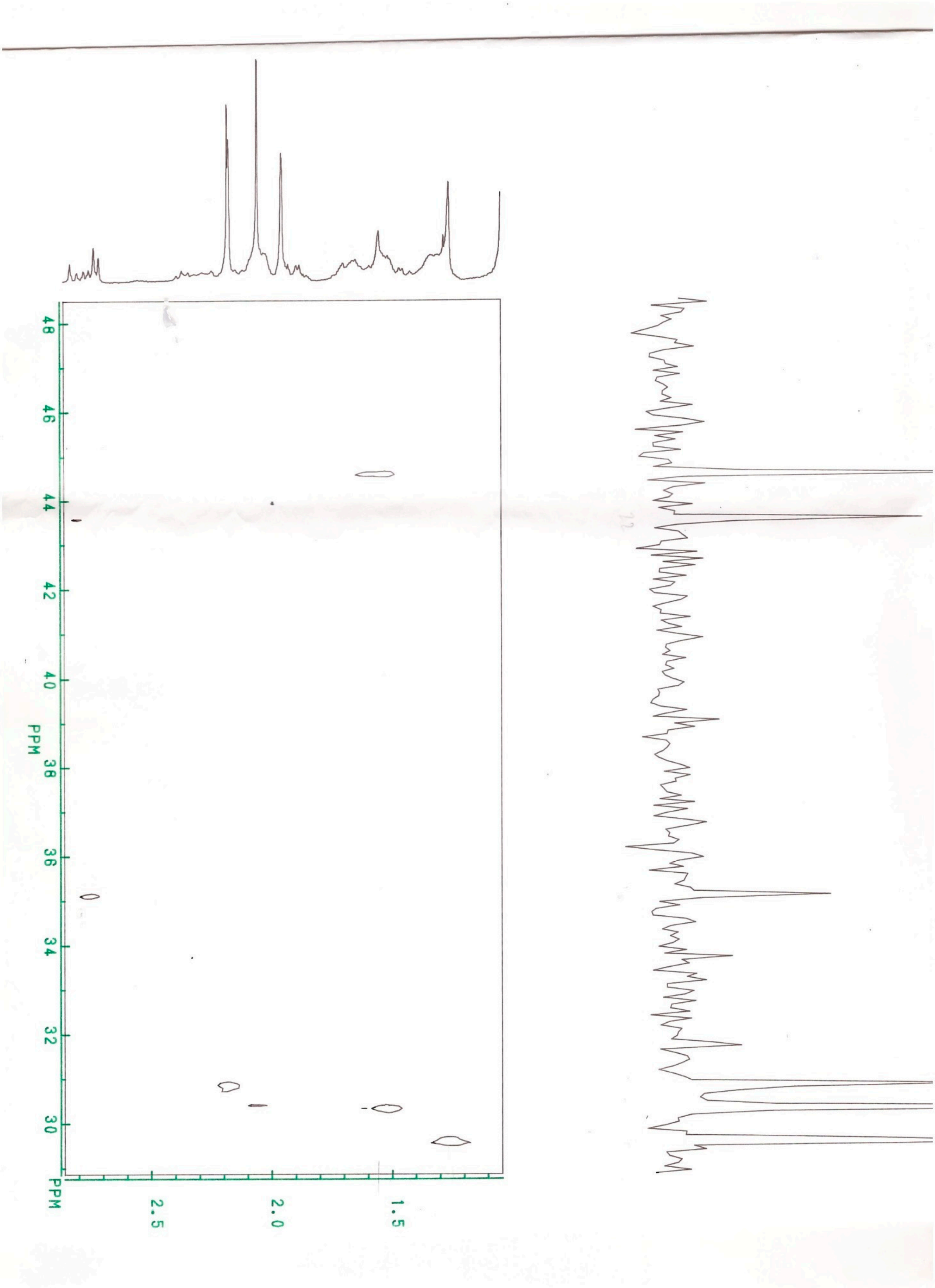

S14A

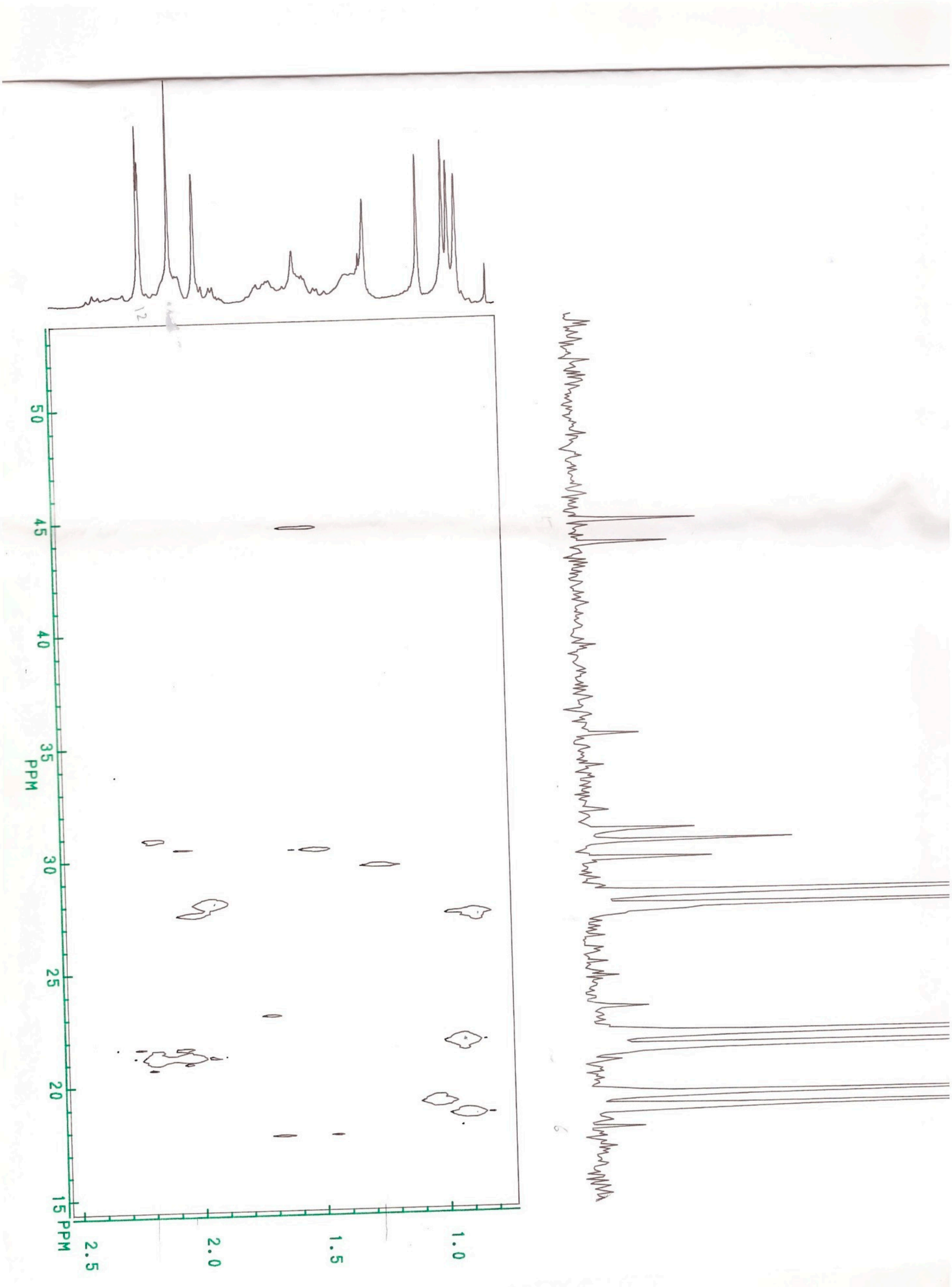

S14B

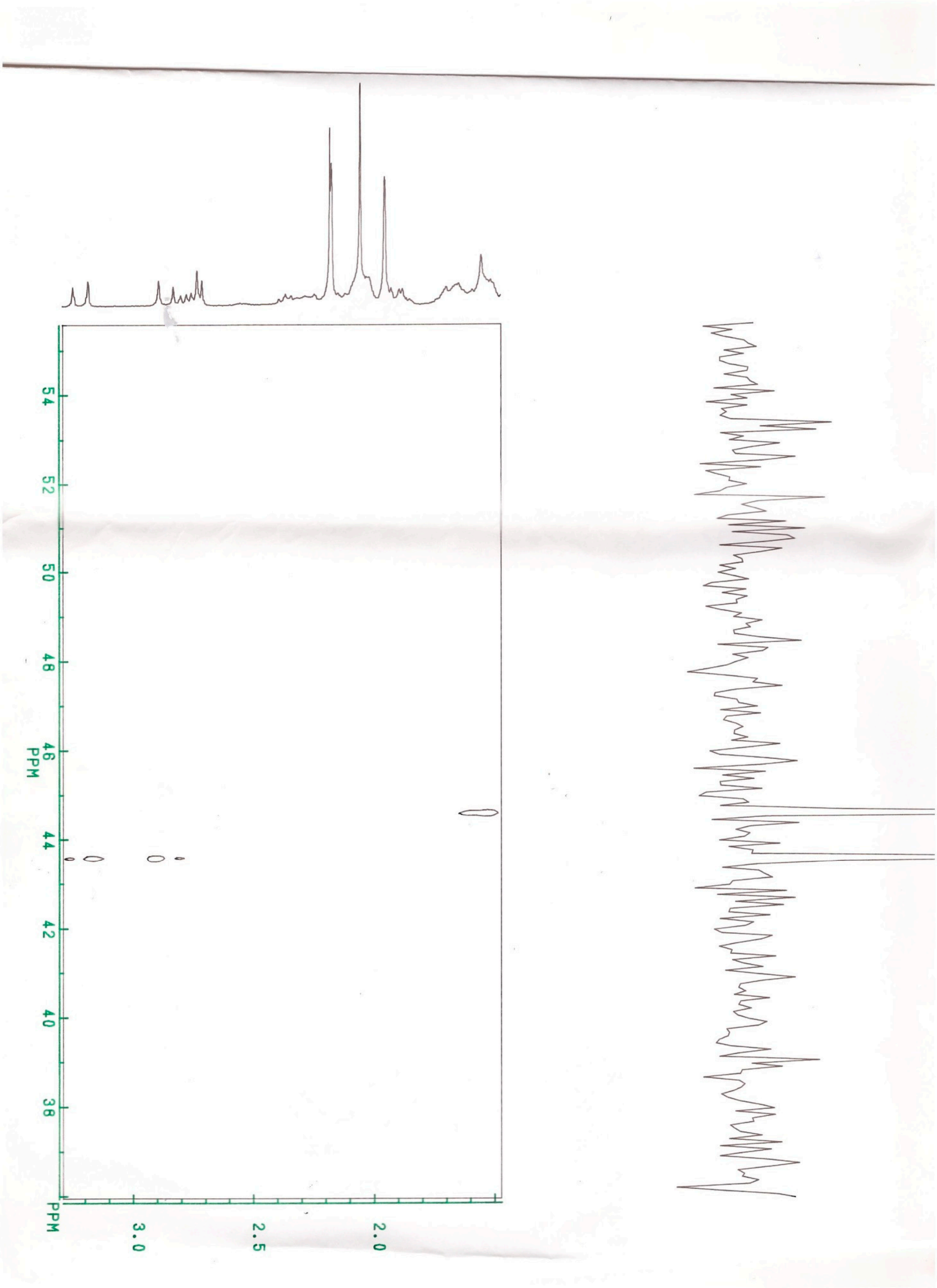

S14C

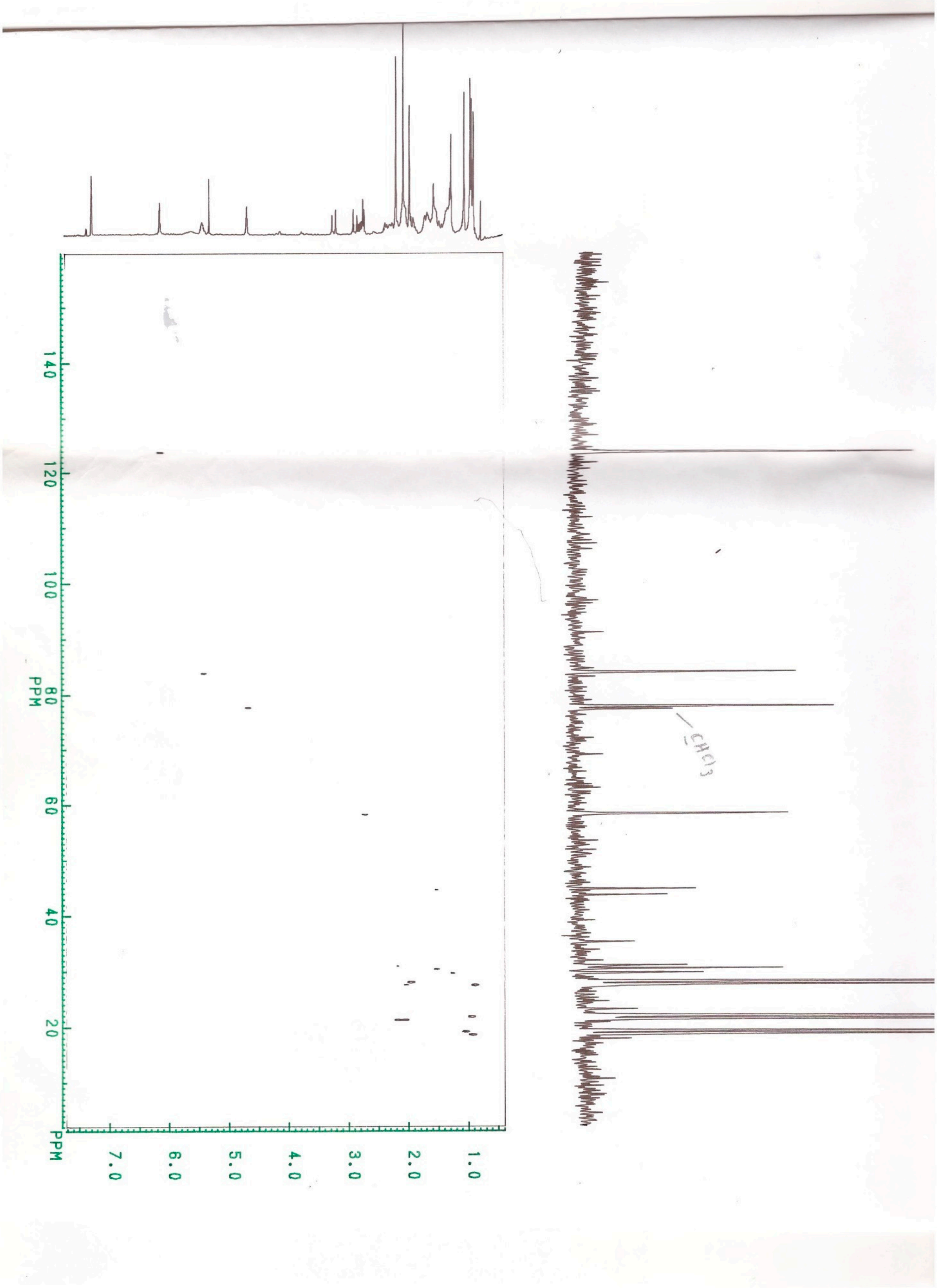

S14D

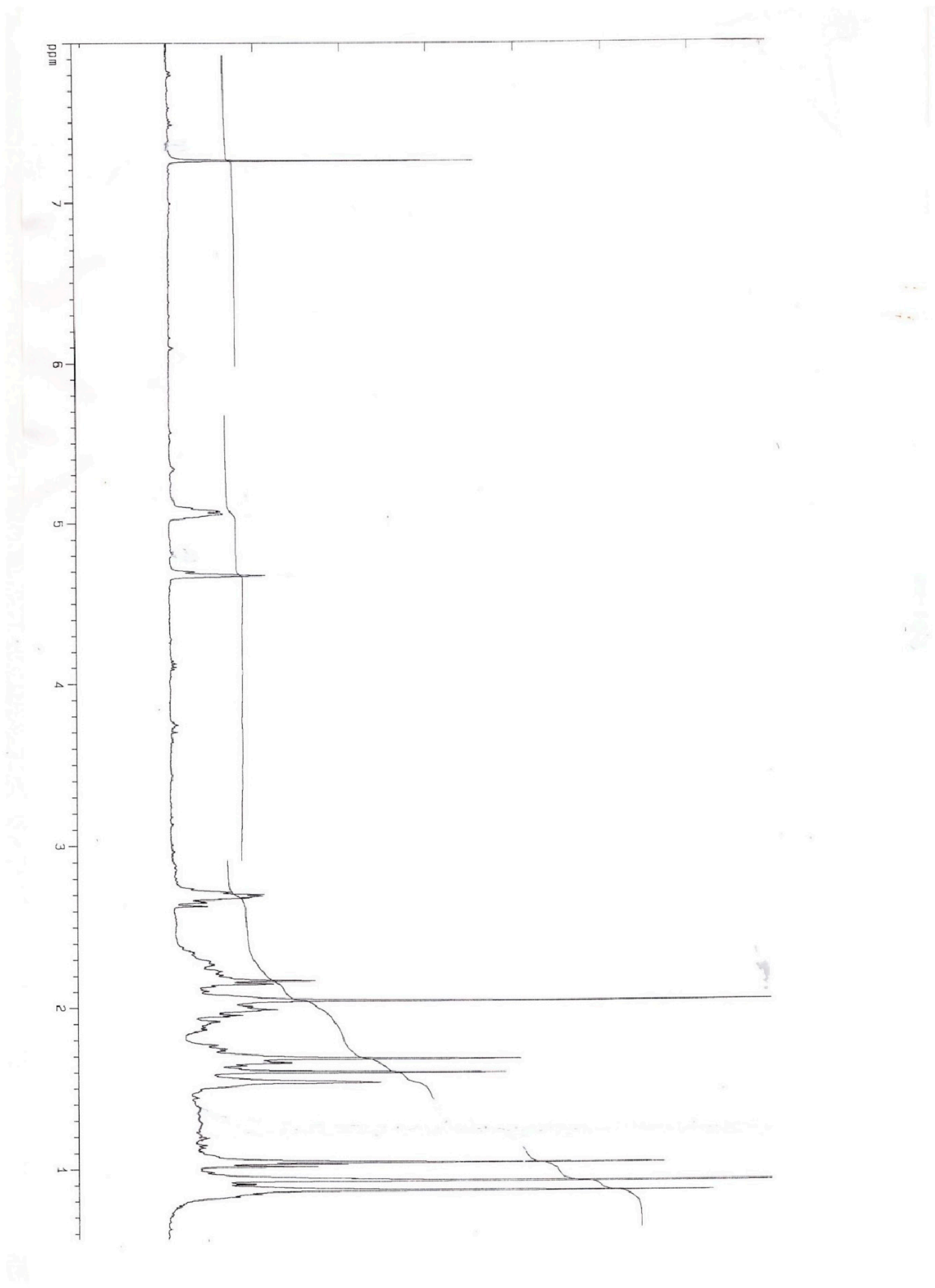
ppm
7
6
5
4
3
2
1

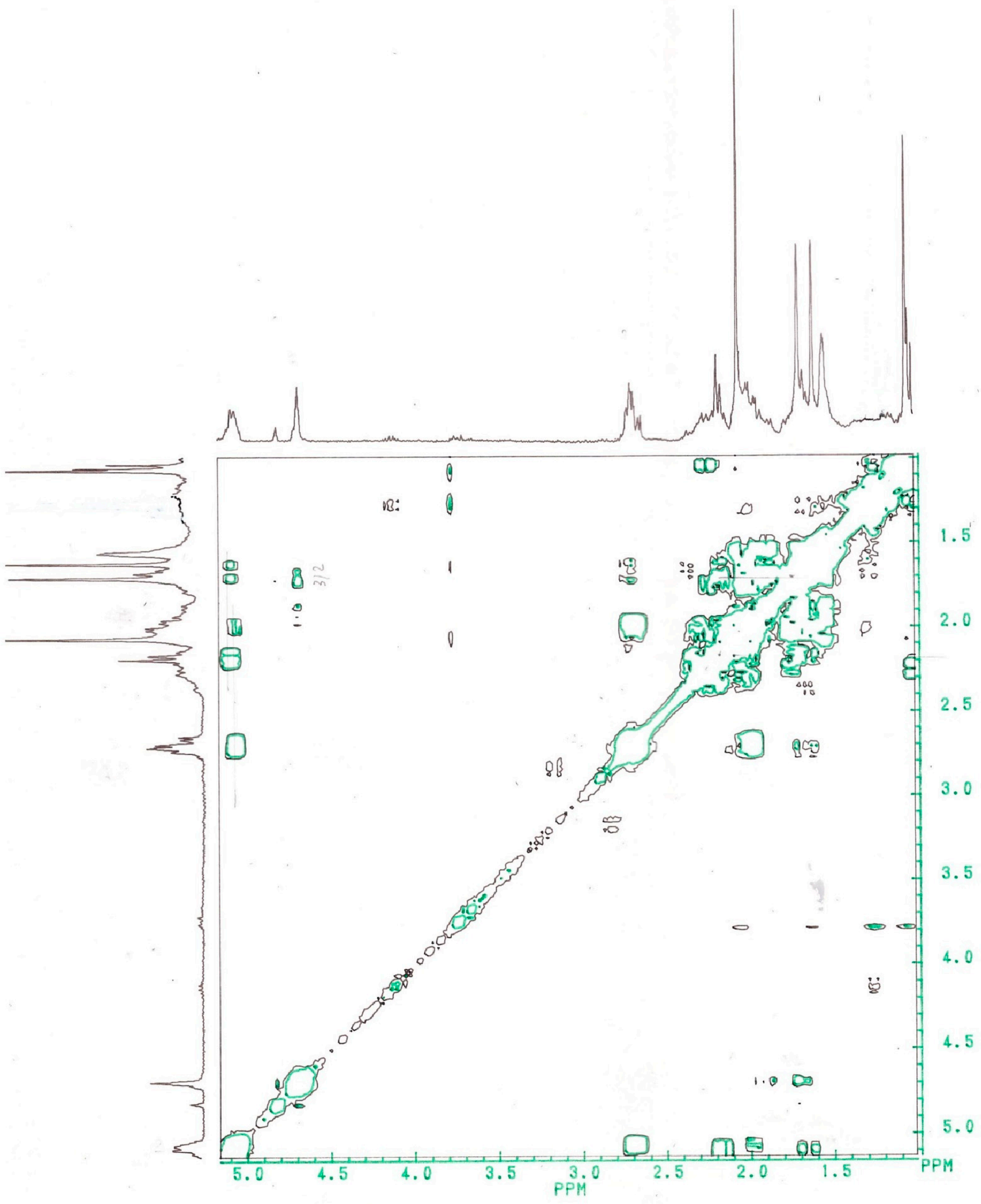

S16A

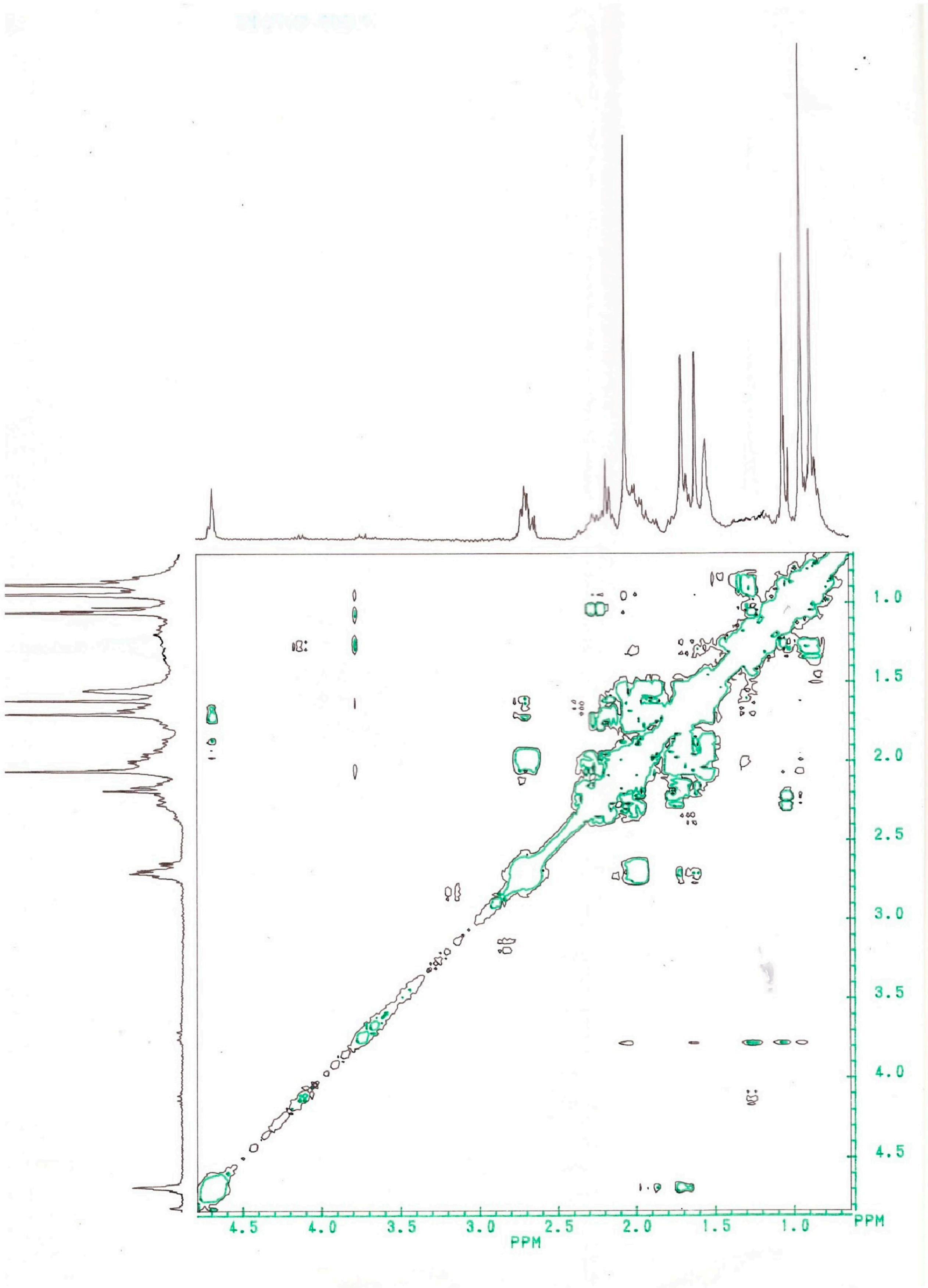

S16B

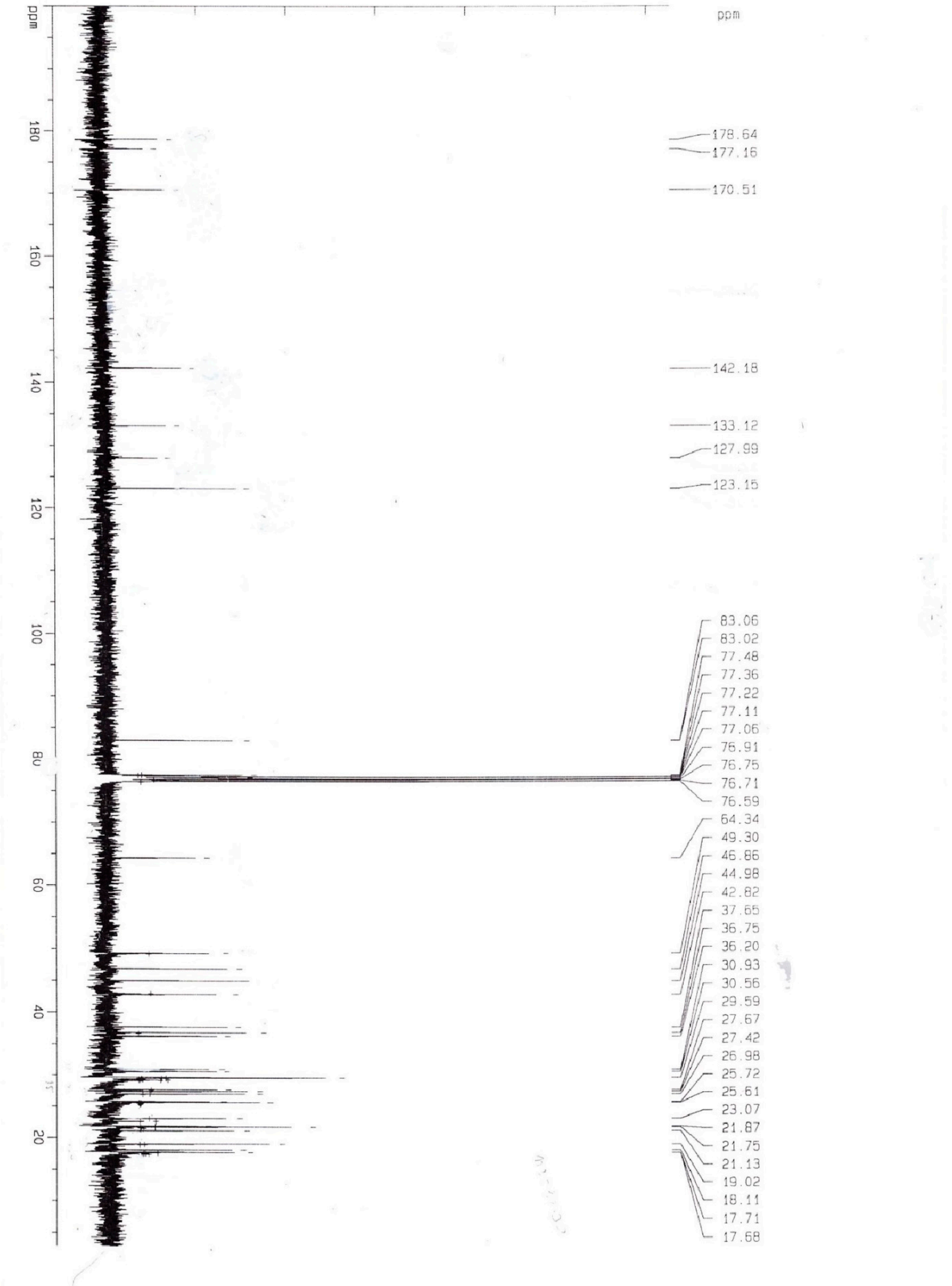
ppm
178.64
177.16
170.51
142.18
133.12
127.99
123.15
83.06
83.02
77.48
77.36
77.22
77.11
77.06
76.91
76.75
76.71
76.59
64.34
49.30
46.86
44.98
42.82
37.65
36.75
36.20
30.93
30.56
29.59
27.67
27.42
26.98
25.72
25.61
23.07
21.87
21.75
21.13
19.02
18.11
17.71
17.68
180
160
140
120
100
80
60
40
20
ppm

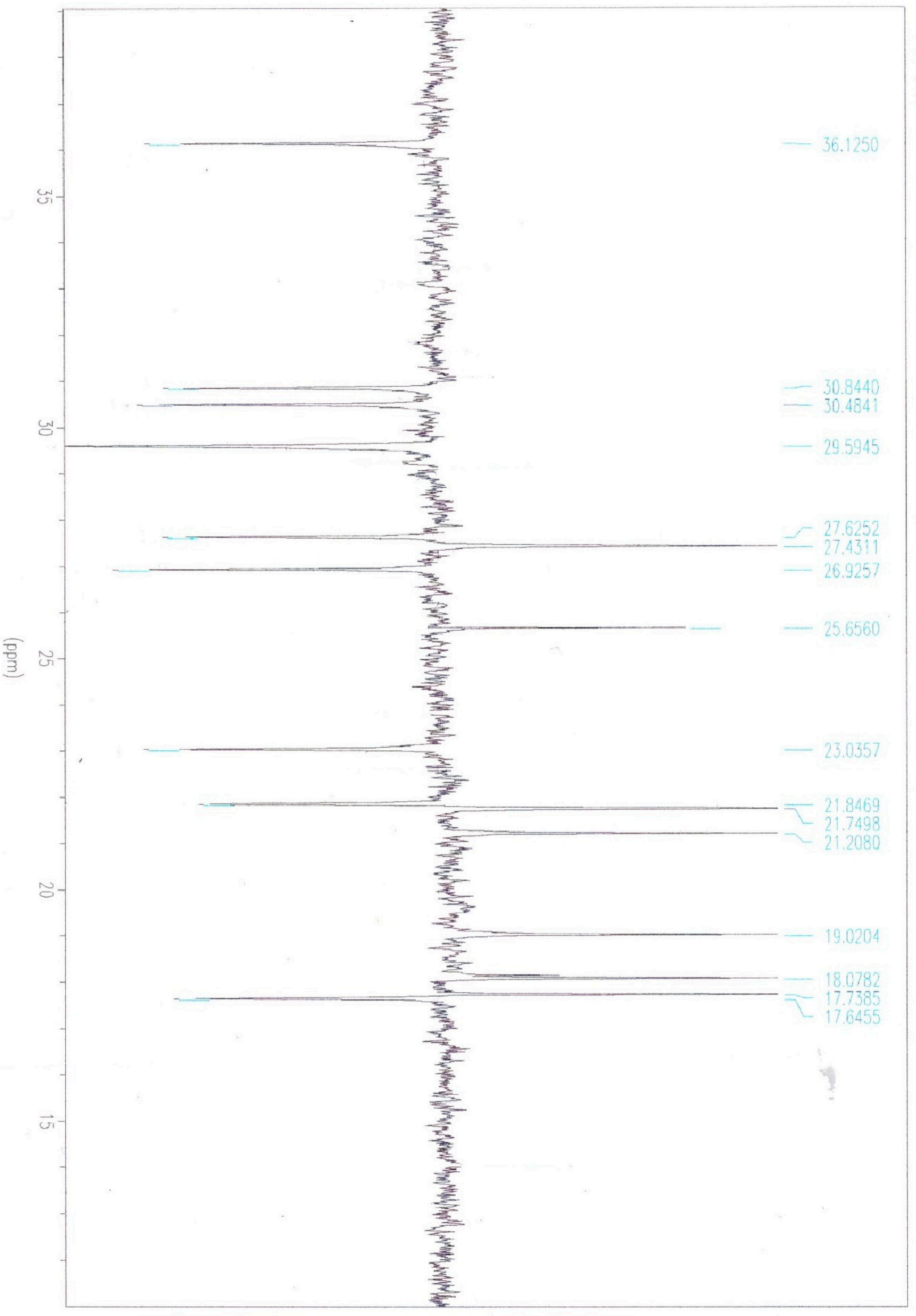
36.1250
30.8440
30.4841
29.5945
27.6252
27.4311
26.9257
25.6560
23.0357
21.8469
21.7498
21.2080
19.0204
18.0782
17.7385
17.6455
35
30
25
(ppm)
20
15

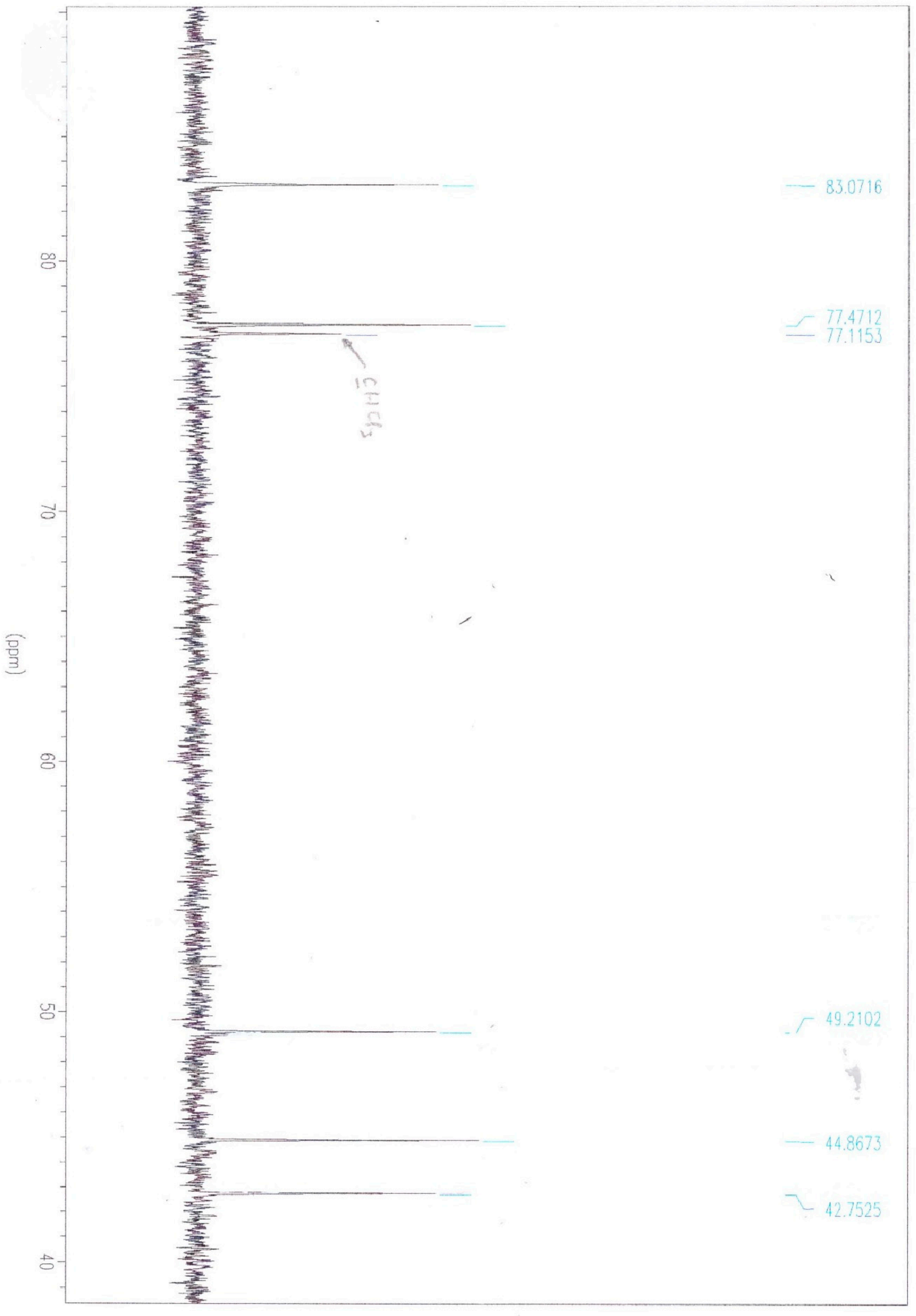

83.0716
77.4712
77.1153
CHCl3
49.2102
44.8673
42.7525
80
70
60
50
40
(ppm)

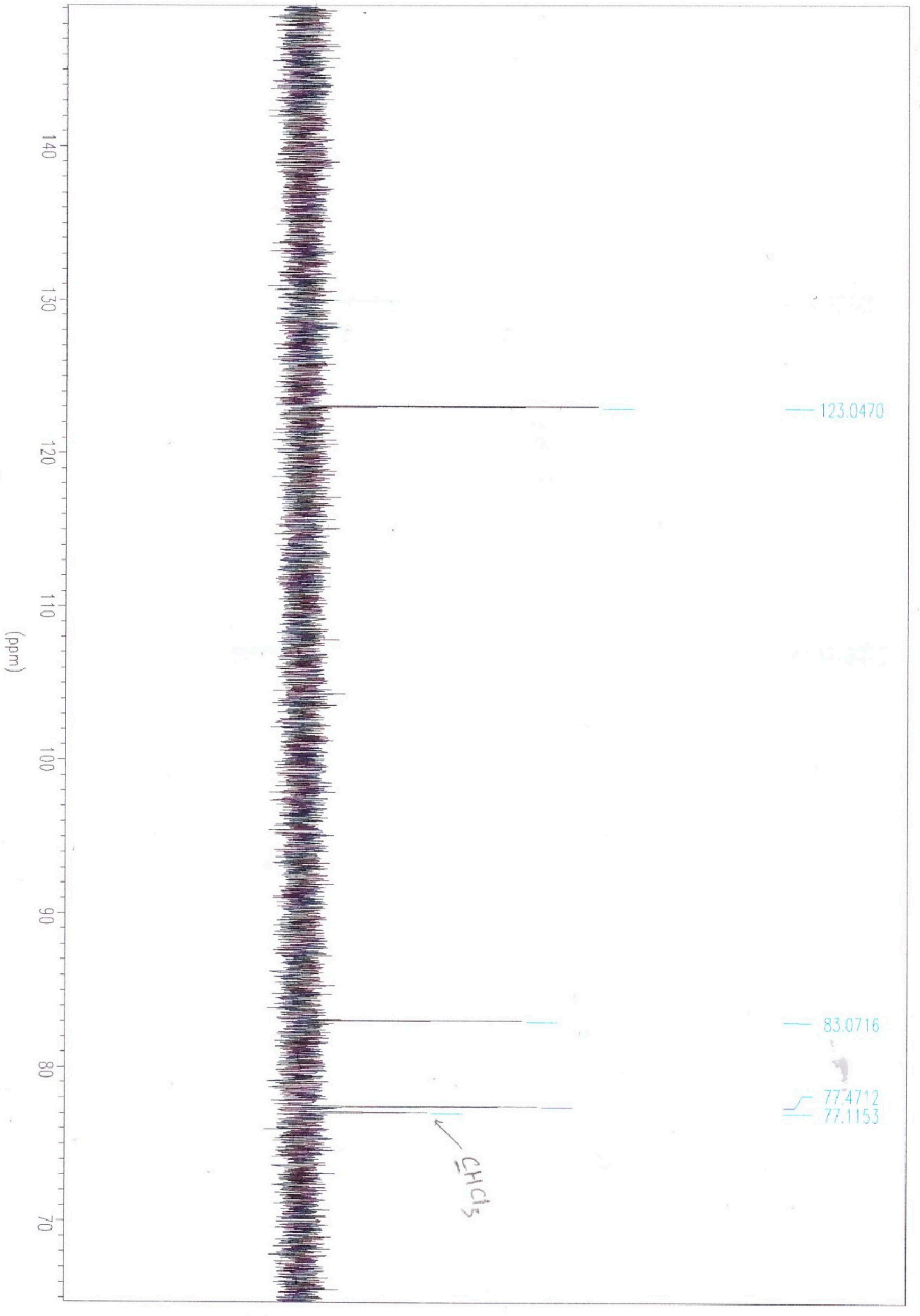
140
130
120
110
(ppm)
100
90
80
70
123.0470
83.0716
77.4712
77.1153
CHCl3

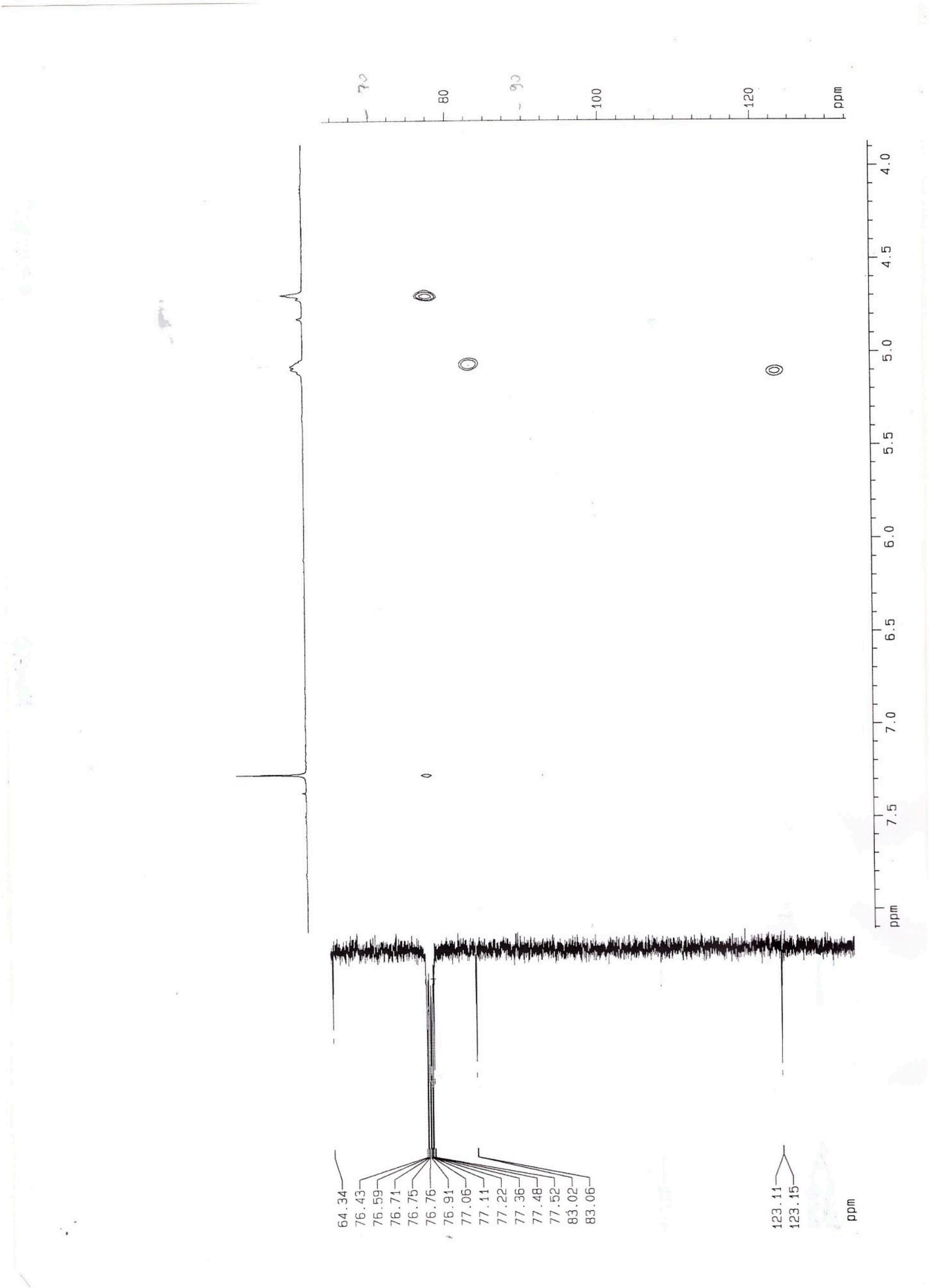

80
100
120
ppm
4.0
4.5
5.0
5.5
6.0
6.5
7.0
7.5
ppm
64.34
76.43
76.59
76.71
76.75
76.76
76.91
77.06
77.11
77.22
77.36
77.48
77.52
83.02
83.06
123.11
123.15
ppm

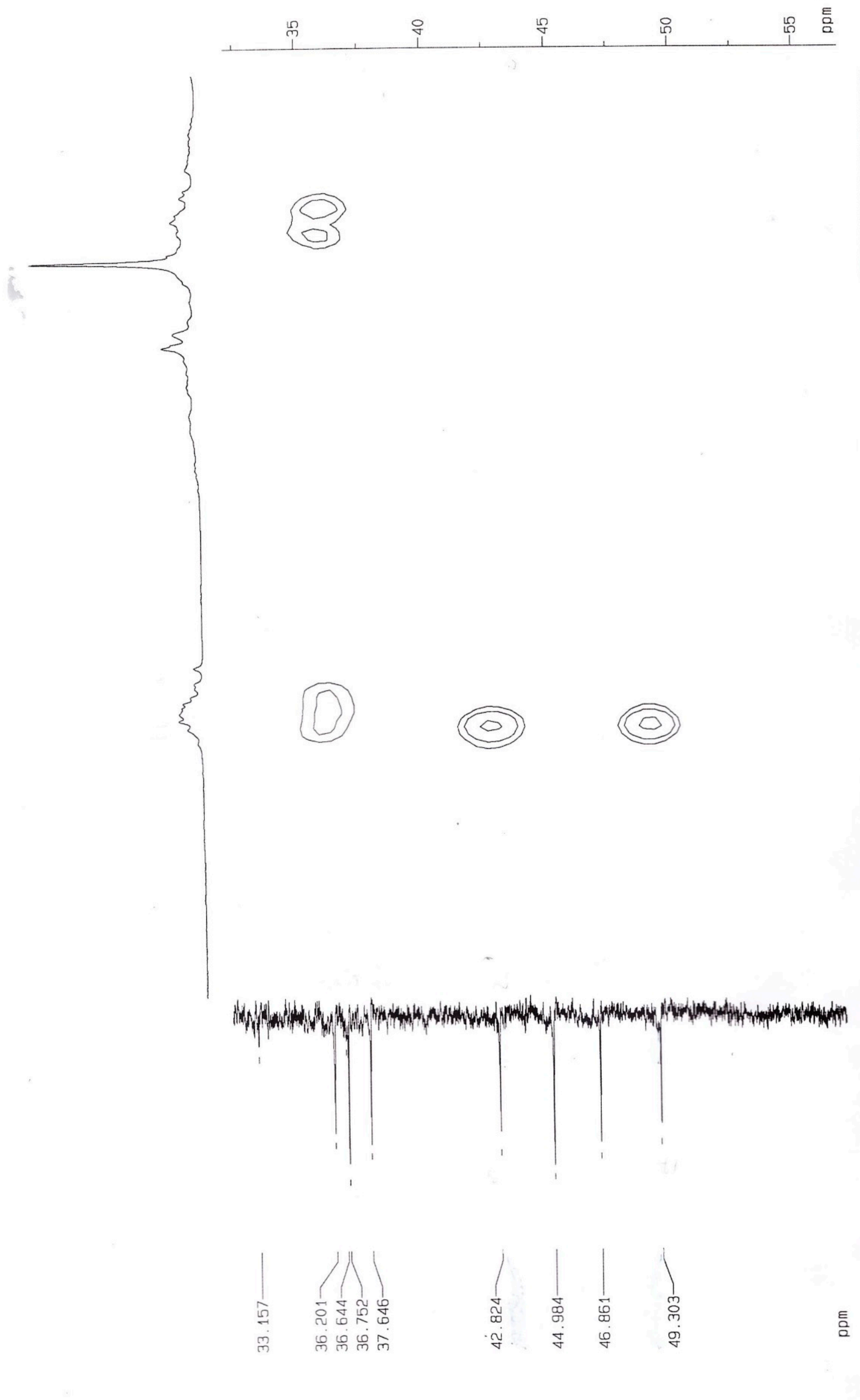
35
40
45
50
55
ppm
2.0
2.2
2.4
2.6
2.8
3.0
ppm
33.157
36.201
36.644
36.752
37.646
42.824
44.984
46.861
49.303
ppm

S19B

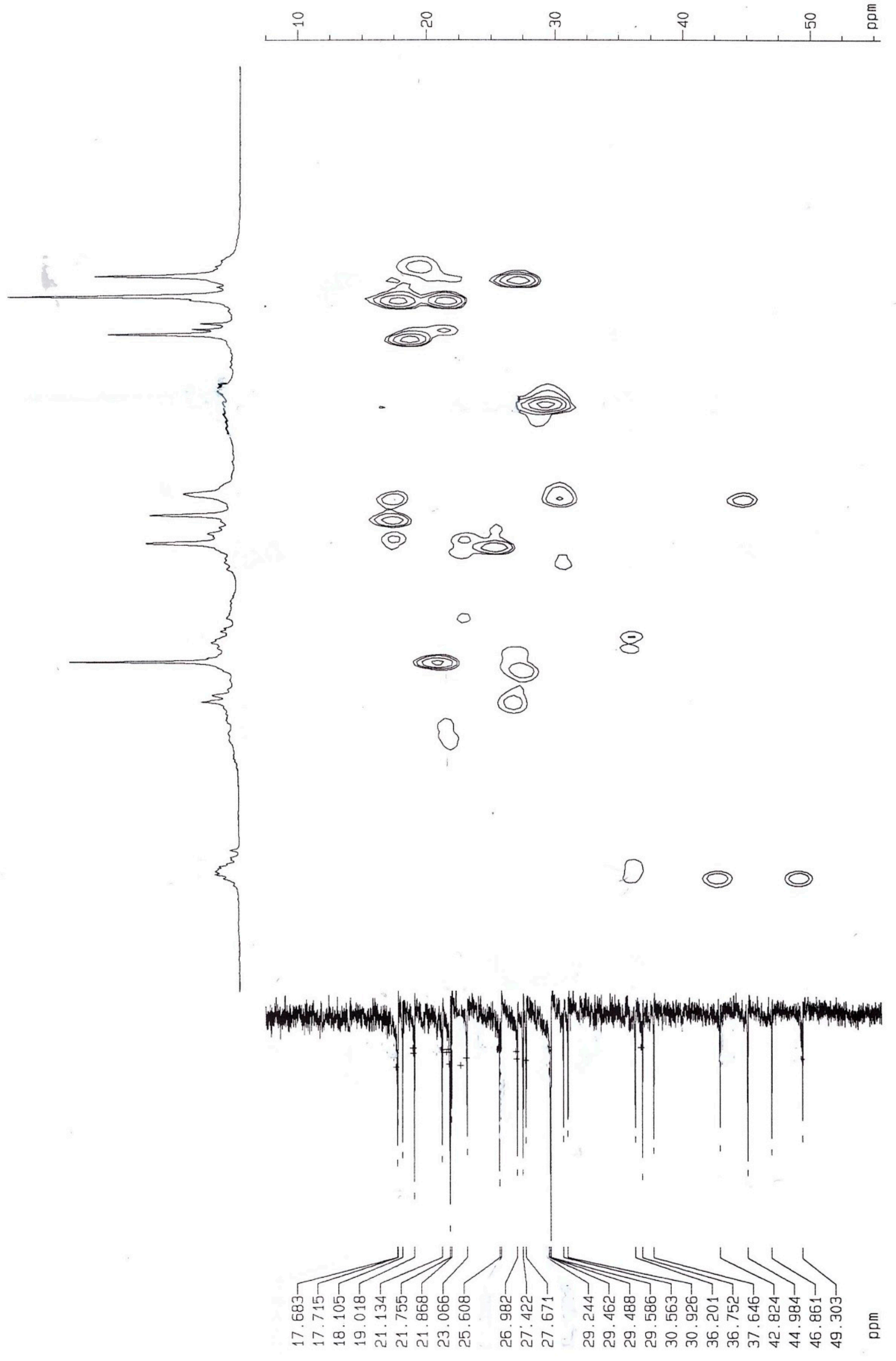

10
20
30
40
50
ppm
0.5
1.0
1.5
2.0
2.5
ppm
17.683
17.715
18.105
19.018
21.134
21.755
21.868
23.066
25.608
26.982
27.422
27.671
29.244
29.462
29.488
29.586
30.563
30.926
36.201
36.752
37.646
42.824
44.984
46.861
49.303
ppm

S19C
